# Effects of urban functional fragmentation on nitrogen dioxide (NO_2_) variation with anthropogenic-emission restriction in China

**DOI:** 10.1038/s41598-021-91236-w

**Published:** 2021-06-07

**Authors:** Yuan Meng, Man Sing Wong, Hanfa Xing, Rui Zhu, Kai Qin, Mei-Po Kwan, Kwon Ho Lee, Coco Yin Tung Kwok, Hon Li

**Affiliations:** 1 Department of Land Surveying and Geo-Informatics, The Hong Kong Polytechnic University, Hung Hom, Hong Kong; 2 Research Institute for Sustainable Urban Development, The Hong Kong Polytechnic University, Hung Hom, Hong Kong; 3 School of Geography, South China Normal University, Guangzhou, Guangdong China; 4 College of Geography and Environment, Shandong Normal University, Jinan, Shandong China; 5 School of Environment and Spatial Informatics, China University of Mining and Technology, Xuzhou, China; 6 Department of Geography and Resource Management, and Institute of Space and Earth Information Science, The Chinese University of Hong Kong, Sha Tin, Hong Kong; 7 Department of Human Geography and Spatial Planning, Utrecht University, 3584 CB Utrecht, The Netherlands; 8 Department of Atmospheric and Environmental Sciences, Gangneung-Wonju National University, Gangneung, 25457 South Korea

**Keywords:** Sustainability, Environmental impact

## Abstract

Urban functional fragmentation plays an important role in assessing Nitrogen Dioxide (NO_2_) emissions and variations. While the mediated impact of anthropogenic-emission restriction has not been comprehensively discussed, the lockdown response to the novel coronavirus disease 2019 (COVID-19) provides an unprecedented opportunity to meet this goal. This study proposes a new idea to explore the effects of urban functional fragmentation on NO_2_ variation with anthropogenic-emission restriction in China. First, NO_2_ variations are quantified by an Autoregressive Integrated Moving Average with external variables-Dynamic Time Warping (SARIMAX-DTW)-based model. Then, urban functional fragmentation indices including industrial/public Edge Density (ED) and Landscape Shape Index (LSI), urban functional Aggregation Index (AI) and Number of Patches (NP) are developed. Finally, the mediated impacts of anthropogenic-emission restriction are assessed by evaluating the fragmentation-NO_2_ variation association before and during the lockdown during COVID-19. The findings reveal negative effects of industrial ED, public LSI, urban functional AI and NP and positive effects of public ED and industrial LSI on NO_2_ variation based on the restricted anthropogenic emissions. By comparing the association analysis before and during lockdown, the mediated impact of anthropogenic-emission restriction is revealed to partially increase the effect of industrial ED, industrial LSI, public LSI, urban functional AI and NP and decrease the effect of public ED on NO_2_ variation. This study provides scientific findings for redesigning the urban environment in related to the urban functional configuration to mitigating the air pollution, ultimately developing sustainable societies.

## Introduction

Urban functional fragmentation refers to the breaking up of urban functional areas, such as residential and industrial lands, into more isolated segments^1,2^. It encourages diverse human activities such as vehicular mobility and large quantities of manufactures, result in the diversity and vulnerable fractions of urban function areas and cause a series of anthropogenic pollution such as noise and air pollutants^3,4^. Nitrogen Dioxide (NO_2_) has been considered as one of the major anthropogenic emissions^5,6^, which is associated with several social environmental issues, such as cardiopulmonary mortality^7^, lung cancer^8^ and severe air pollution^9^. The anthropogenic emissions of NO_2_ are mainly attributed by fossil fuel uses from various urban functions such as traffic, industrial and public uses^10^. Evaluating the impact of urban functional fragmentation is essential to estimate NO_2_ emissions.

The rapid emergence of the novel coronavirus disease 2019 (COVID-19) has significantly changed the business-as-usual circumstance of the anthropogenic NO_2_ emissions^11^. Due to the policies of lockdowns and restricted social distancing proposed by the governments, the regular socioeconomic activities are drastically reduced^12^, leading to the variation of anthropogenic-generated emissions^13–15^. These changes have brought opportunities to estimate the impact of urban functional fragmentation under different circumstances of NO_2_ emissions, i.e. the differences between NO_2_ emissions before and after lockdowns^16,17^. For instance, the lockdowns have significantly reduced the traffic flows, which tend to exceed the road capability before lockdowns, and thus change the influence of roads with heavy traffics on NO_2_ emissions^18–20^.

Current studies on the association analysis between urban functional fragmentation and NO_2_ concentration can be reviewed based on three main aspects, including the air pollution metrics, fragmentation metrics and the methods for association analysis. For quantifying NO_2_ emissions, existing approaches can be divided into two aspects, including the total column-based and difference-based quantification. The total column-based NO_2_ quantification, involving hourly, daily, monthly and annual concentration, has been utilized to depict the temporal variation of NO_2_^21–23^. For instance, Li, et al.^23^ proposed multiple dimensions including hourly average values, daily average values and the standard deviation of the peak hours to depict the variation of NO2 concentrations. However, such approach only quantifies the NO_2_ changes within the research period and cannot evaluate the historical NO_2_ trends that may be drastically different from the research-period trends caused by unprecedent events, i.e. COVID-19. To fill this gap, difference-based quantification has been utilized to depict such historical-research period changes of COVID-19. For instance, Venter, et al.^16^ defined the NO_2_ differential as the difference between the NO_2_ concentration in the research period and the average values of the historical three-year baseline.

For the fragmentation metrics for depicting urban functions, both specific metric and urban functions that are highly associated with anthropogenic air pollutant emissions are concerned. In particular, basic quantifications of urban functional areas, including edge length, patch areas, and their synthetical characteristics of fragmentation such as landscape shape index (LSI) and Aggregation Index (AI), Percentage of Like Adjacencies (PLADJ), Number of Patches (NP), Patch Cohesion Index (COHESION), impervious area-weighted mean shape index and contiguity index, have been utilized to estimate the variations of air pollutants^22,24–27^. Moreover, research has indicated that industrial and public functions, as well as the mixture of urban functions, which are highly related to the use of energy resources such as fuels, minerals and electric power, show a higher impact on anthropogenic air pollution emissions^28–30^. He, et al.^31^ have indicated the positive trends between the industrial functional fragmentation and NO_2_ concentrations. Research has also revealed that the distribution of public urban functions including greenery and parks show the potential to influence NO_2_ variations^32,33^.

For association analysis, regression models such as ordinary least squares, spatial autoregressive model and panel data model have been considered. Lee^34^ adopted ordinary least squares and two-level regression models to control geographic and metropolitan-level socioeconomic factors for urban form estimation, and further indicated that high-level urban function mixing shows the potential to reduce air pollutant emissions. Li and Zhou^35^ involved the spatial correlation of urban fragmentation characteristics and applied the spatial autoregressive model, suggesting that scattered polycentric cities in China are associated with better air quality. Panel data analysis has also been utilized to quantify the link between urban forms and air pollution, revealing negative association between urban fragmentation and air quality^36^.

Despite of the above discussed approaches in existing studies, limitations still exist faced with the emergence of COVID-19^37–40^. First, although extracting the average value in the difference-based quantification concerns the historical NO_2_ variation, the changes of the historical NO_2_ concentration is ignored. For instance, the three-year average NO_2_ values cannot depict the potential changes among these years. Thus, effective approaches of quantifying the overall temporal trends of NO_2_ concentration is required. In addition, the lag effect should be considered in the difference-based quantification to avoid the biases caused by the point-to-point (such as day-to-day measurement) differential calculation. Second, considering that most human activities including business and leisure have been significantly changed by the COVID-19, the fragmentation depiction should focus on the industrial and public related urban functions. The synthetical urban functional characteristics also need be concerned to implement the overall urban functional fragmentation quantification. Third, despite that multi-perspective covariates such as meteorological and spatial factors have been concerned in previous research, different scenarios of anthropogenic emissions during COVID-19 caused by the lockdown policies are not fully considered in evaluating the impact of urban functional fragmentation.

Meanwhile, among the countries and regions for analysis, China has experienced drastic industrialization and urbanization^41,42^, leading to increasing urban population and gross domestic production among cities^43,44^. During this process, urban landscapes and configurations in China have changed dramatically to improve urbanization process^45,46^. Existing studies have found that this rapid transformation of urban forms in China is highly associated with air pollution emissions^47^. Recently, due to the COVID-19, the coronavirus quarantine proposed in China has led to economic slowdown^48^, causing drastic decline of anthropogenic emissions and the variation of NO_2_ than ever before^49,50^. The urbanization process and NO_2_ changes in the lockdown period in China have provided opportunities to explore the impact of urban functional fragmentation on NO_2_ variations based on different scenarios of anthropogenic emissions.

To fill with these gaps, this paper investigates the impact of urban functional fragmentation on NO_2_ variation mediated by the changes of anthropogenic air pollutant emissions in China during COVID-19. The objective of this study is (1) to quantify the differential of temporal NO_2_ during COVID-19 compared with NO_2_ in normal days based on the historical NO_2_ trends and the potential lag effects; (2) to depict both single-functional and synthetical-functional fragmentation characteristics driven by the changes of human activities; and (3) to investigate the association between urban functional fragmentation and NO_2_ variation based on the different scenarios of anthropogenic emissions during COVID-19.

**Figure 1:**
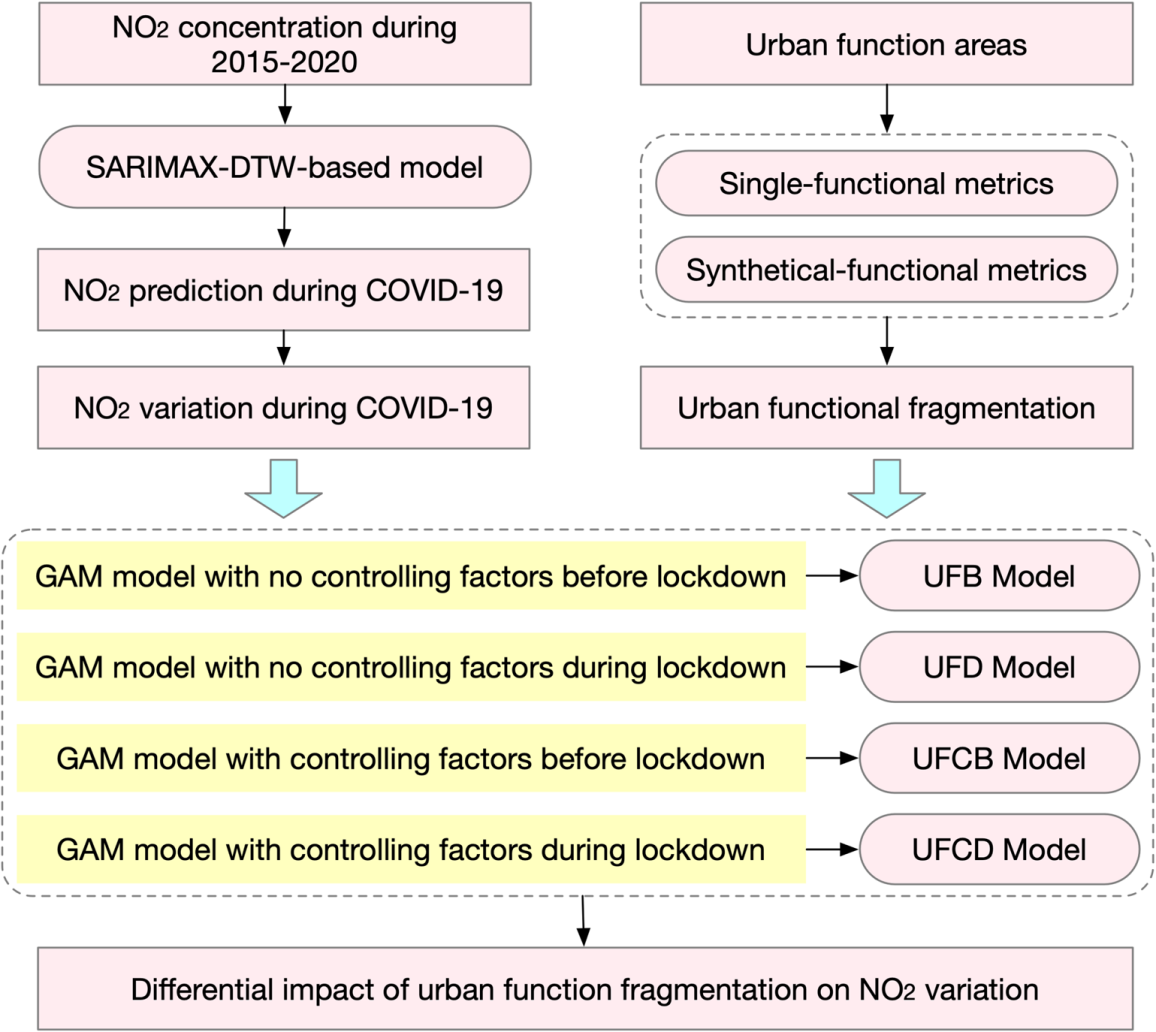
The overview framework. Three components are involved, SARIMAX-DTW-based model for NO_2_ variation estimation, human-activity-driven urban functional fragmentation quantification and UFB, UFD, UFCB and UFCD Models for association analysis.

To achieve the objectives, a new research framework is proposed as follows (Fig. 1). First, an Autoregressive Integrated Moving Average with external variables-Dynamic Time Warping (SARIMAX-DTW)-based model is proposed to quantify NO_2_ variations. Then, human activity-driven metrics are considered, including industrial/public Edge Density (ED) and LSI and urban functional AI and NP, to depict fragmentation characteristics of urban functions. Finally, to different scenarios of NO_2_ emissions, four models including Urban functional Fragmentation characteristics Before lockdowns (UFB)-based model, Urban functional Fragmentation characteristics During lockdowns (UFD)-based model, Urban functional Fragmentation characteristics and Controlling variables Before lockdowns (UFCB)-based model and Urban functional Fragmentation characteristics and Controlling variables During lockdowns (UFCD)-based model are proposed based on the Generalized Additive Models (GAMs). This study will gain a better understanding of functional fragmentation-NO_2_ variation relationship when considering different scenarios of anthropogenic emissions and will help government provide effective guidelines in policy-making to develop sustainable societies.

## Results

### NO_2_ variation during COVID-19 epidemic

**Figure 2:**
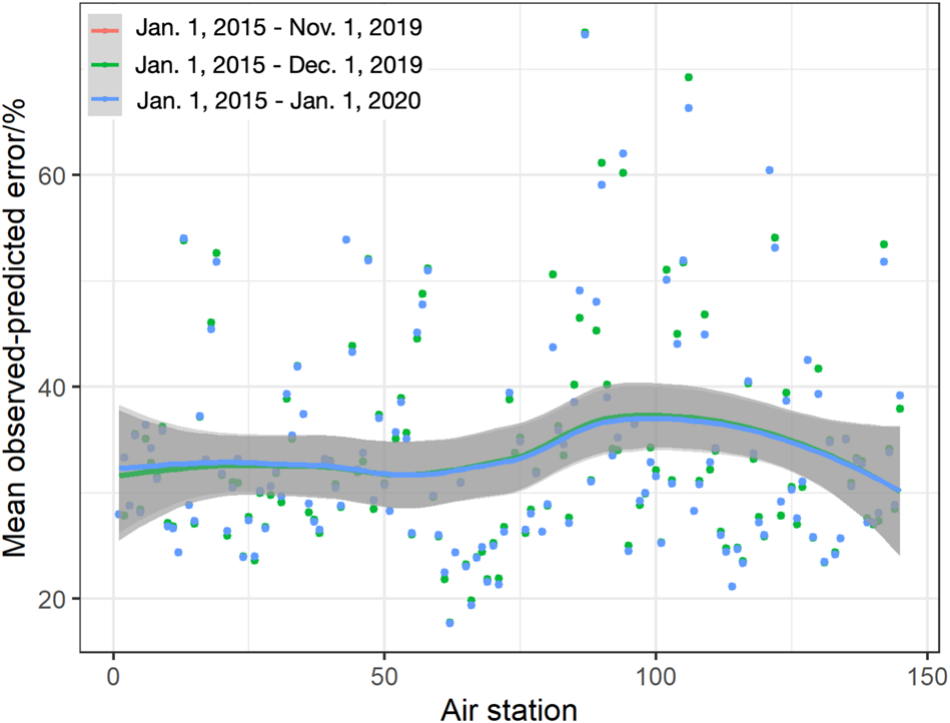
Mean daily observed-predicted NO_2_ errors based on training data from three time periods, including Jan. 1st, 2015 to Nov. 1st, 2019, from Jan. 1st, 2015 to Dec. 1st, 2019 and from Jan. 1st, 2015 to Jan. 1st, 2020, to determine the time period of training data for SARIMAX modelling.

Figure 2 reveals the mean observed-predicted error of NO_2_ prediction in 145 air stations among three time periods, (1) from Jan. 1st, 2015 to Nov. 1st, 2019, (2) from Jan. 1st, 2015 to Dec. 1st, 2019 and (3) from Jan. 1st, 2015 to Jan. 1st, 2020. Despite of the differential in time periods, similar distributions mean observed-predicted error are observed within 20–40%. It indicates that no significant impacts of time period division are shown on the NO_2_ prediction. As we intend to predict NO_2_ concentrations during COVID-19 (from Jan. 2020), NO_2_ from Jan. 1st, 2015 to Jan. 1st, 2020 were chosen for SARIMAX modelling and those from Jan. 1st, 2020 to May. 1st, 2020 were utilized for prediction.

**Figure 3:**
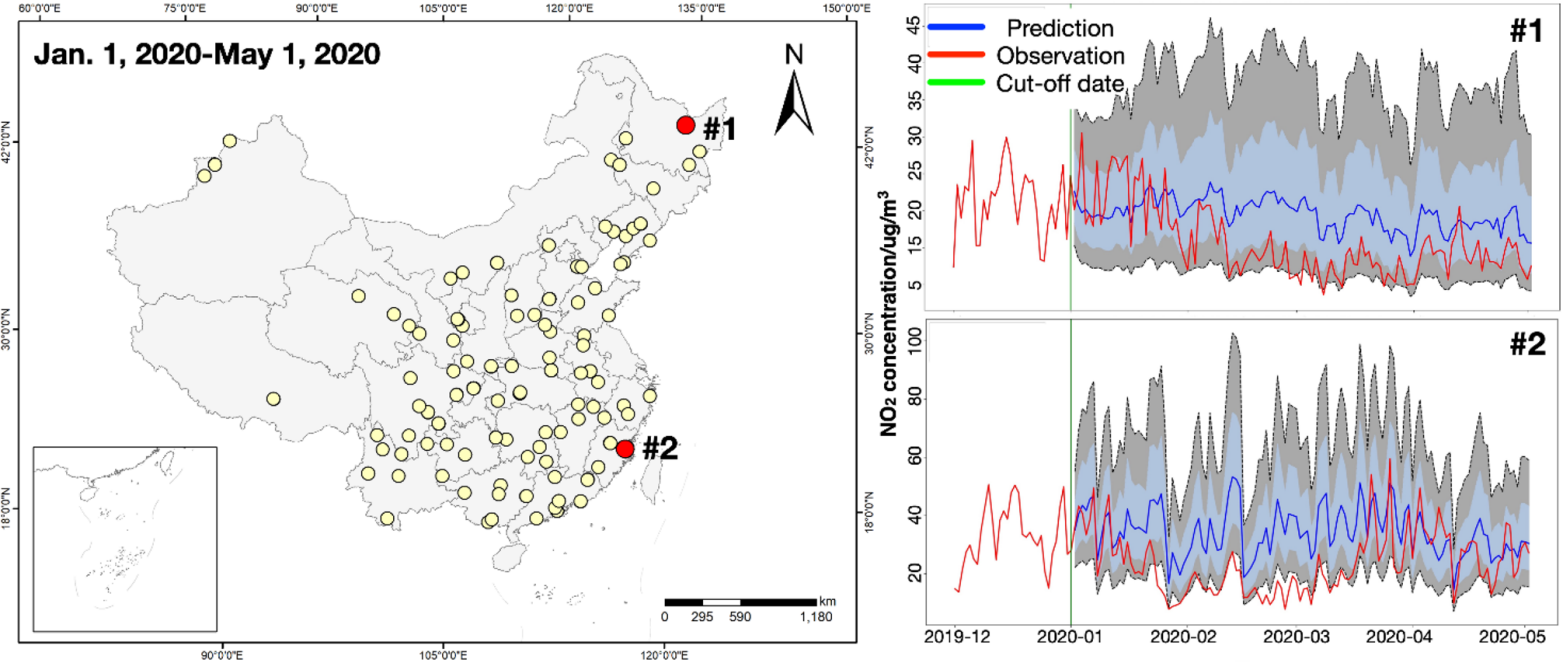
Predicted NO_2_ concentrations of selected air stations using SARIMAX during Jan. 1st, 2020 to May 1st, 2020. Two air stations #1 and #2 were selected. Red lines indicate the observed NO_2_ concentrations, blue lines indicate the predicted NO_2_ concentrations, and green lines represent the cut-off date of NO_2_ estimation. The blue and grey areas indicate the one and two standard deviation(s) of NO_2_ predictions. The map is performed using ArcGIS Pro software (version 2.7, https://www.esri.com/en-us/arcgis/products/arcgis-pro/overview).

On this basis, temporal NO_2_ concentrations in 145 air stations during Jan. 1st, 2020 to May 1st, 2020 were utilized as testing data in SARIMAX models. The prediction of NO_2_ concentration of 145 air stations is shown in Fig. S1. In particular, Fig. 3 displays two selected air stations to illustrate the predicted NO_2_ patterns. Red lines and blue lines represent the overall temporal trends of observed and predicted NO_2_ concentrations, respectively. In particular, the predicted NO_2_ trends are consistent with the periodical patterns of historical NO_2_ before Jan. 1st, 2020. Lower NO_2_ concentrations are shown on the observed NO_2_ trends compared with the predicted ones, which proves the assumption that current lockdown policies show significant impact on transportation restriction, further reduce vehicle emissions and daily NO_2_ concentrations to some extent. These observed-predicted NO_2_ differences are considered as baselines to quantify the overall NO_2_ variation in each air station.

**Figure 4:**
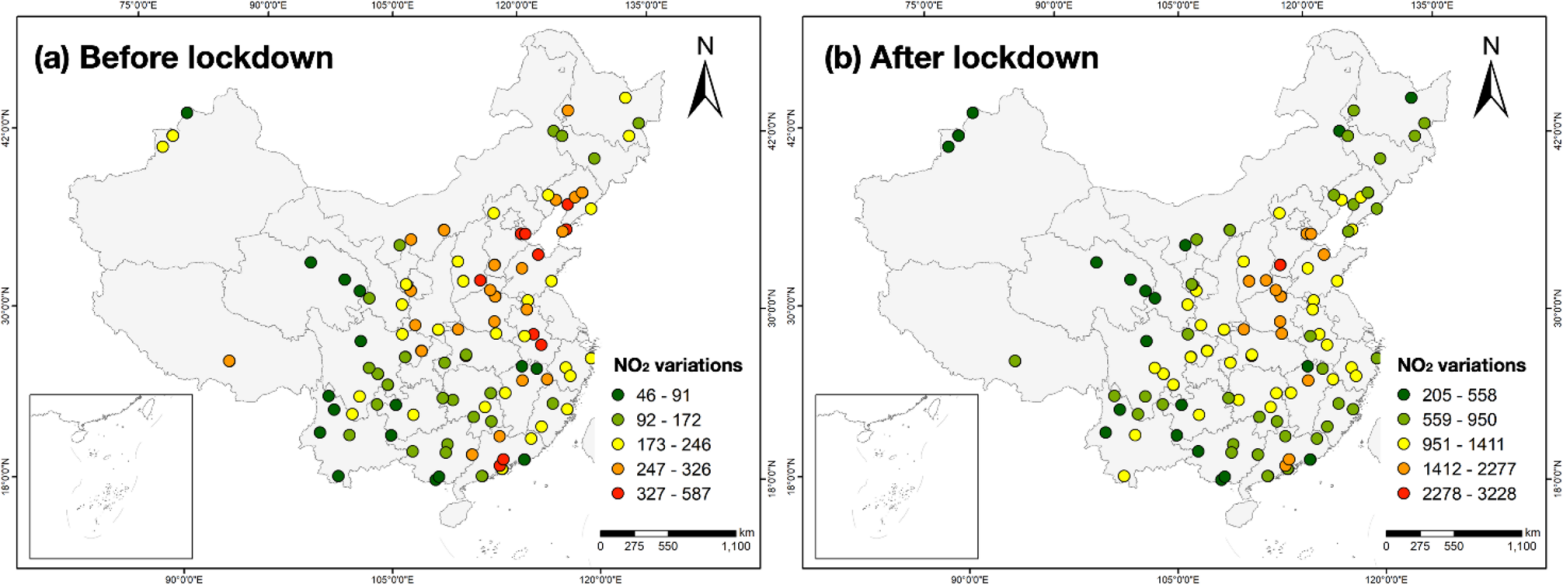
Time-series NO_2_ variations between observed and predicted NO_2_ of 145 air stations before and during lockdown calculated using DTW. (**a**) NO_2_ variations before lockdown; (**b**) NO_2_ variations during lockdown. High values of NO_2_ variations represent greater changes among observed and predicted NO_2_. The maps are performed using ArcGIS Pro software (version 2.7, https://www.esri.com/en-us/arcgis/products/arcgis-pro/overview).

Figure 4 shows the time-series NO_2_ variations values before and during lockdowns in China. The NO_2_ variations during lockdowns are within the range of 205 to 3228, which are much higher than those before lockdowns with variation from 46 to 587. This reveals that emissions caused by urban mobility show less impact during lockdowns, leading to greater NO_2_ variations compared with the period before lockdown. From the perspective of spatial variation, before lockdowns are proposed, most of the air stations with higher NO_2_ variations are located in the eastern and northeastern China, whereas air stations in the southwestern China represent lower differences between observed and predicted NO_2_ concentrations (Fig. 4a). During the lockdowns, the spatial distribution of NO_2_ variations has changed significantly. Specifically, air stations with higher NO_2_ variations mainly distribute in the central east of China, while NO_2_ variation values of the air stations in the north, south and west of China are much lower (Fig. 4b). The detailed quantification of time-series NO_2_ variation is shown in Table S1.

### Urban functional fragmentation

**Figure 5:**
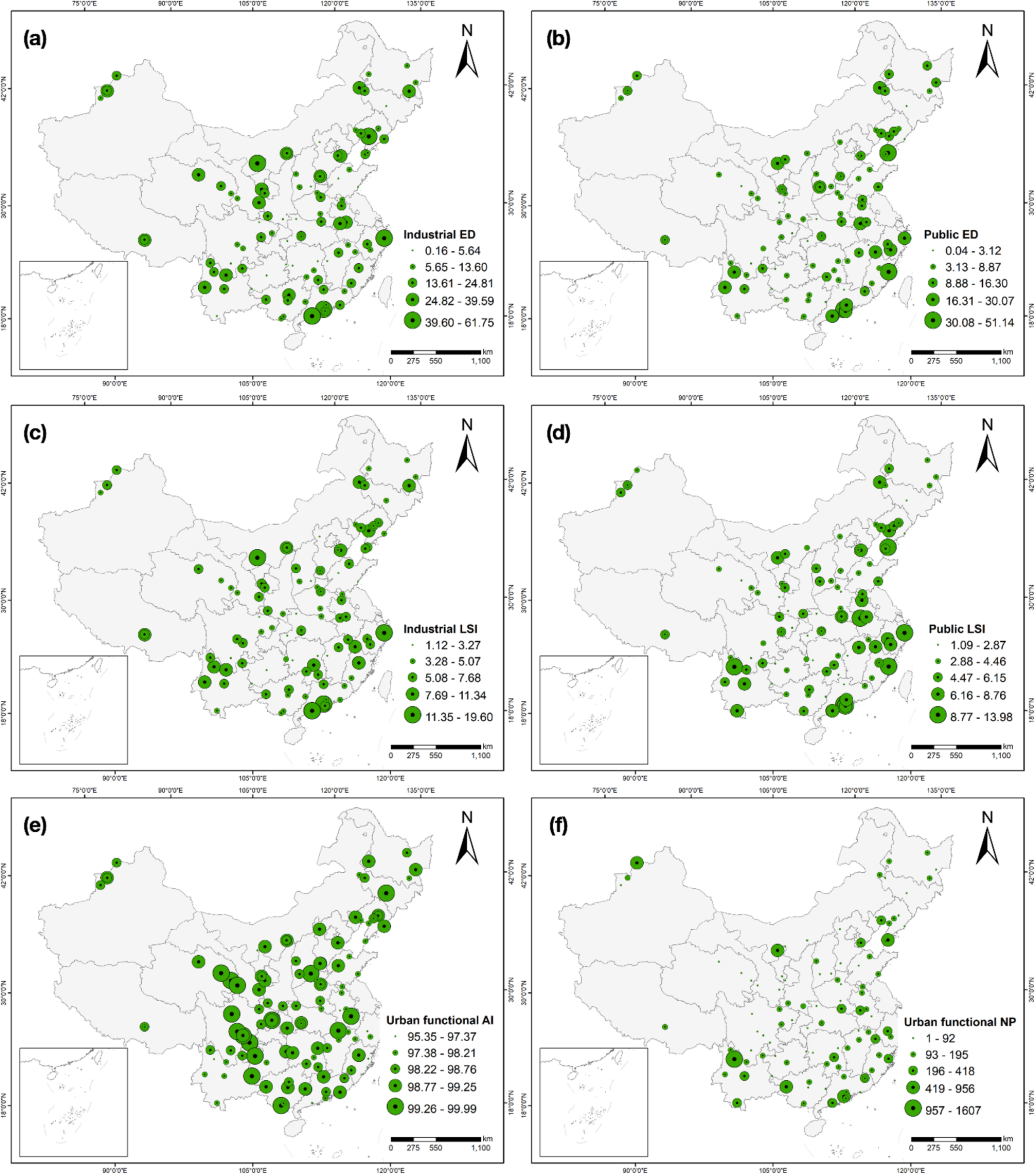
Urban functional fragmentation of each air station. (**a**) Industrial ED; (**b**) Public ED; (**c**) Industrial LSI; (**d**) Public LSI; (**e**) Urban functional AI; (**f**) Urban functional NP. The maps are performed using ArcGIS Pro software (version 2.7, https://www.esri.com/en-us/arcgis/products/arcgis-pro/overview).

Based on the extracted 3 km-radium areas among 145 air stations, the spatial variations of the proposed six urban functional fragmentation characteristics were shown in Fig. 5. The edge densities of industrial function among air stations vary from 0.16 to 61.75. Most of the air stations with higher ED values are located in the southern, eastern and central-north China, while air stations with the lowest ED values distribute in the central-south China (Fig. 5a). The spatial distributions of public-functional ED are much different, with higher values observed in the north and southeast coastal areas of China and lower values in western and central regions (Fig. 5b). For the depicted industrial LSI, air stations denoting significant higher values are located in the southeastern China, while high-industrial LSI-stations distribute in central and northwest China (Fig. 5c). The spatial pattern of public-functional LSI is similar to the public ED characteristic, with higher degree of complexities distributing in eastern and southwestern China (Fig. 5d). Quantified AI of overall urban functions vary from 95.35 to 99.99, with higher values observed in most of the air stations (Fig. 5e). Spatial patterns of urban functional NP are much different compared with urban functional AI, with solely several stations with higher NP values locating sparsely across China (Fig. 5f). The detailed quantification of urban functional fragmentation of 145 air stations is shown in Table S2.

### Effect analysis

Before the regression models were proposed, the multicollinearity among variables should be tested. In particular, the Variance Inflation Factor (VIF) was utilized to estimate the multicollinearity of urban functional fragmentation variables. The results of VIF values are all lower than 10, indicating that multicollinearity is not high among the variables in the proposed models. On this basis, four regression models were proposed to analyze the effect of urban functional fragmentation on NO_2_ variation, including UFB Model without controlling variables before lockdowns, UFD Model without controlling variables during lockdowns, UFCB Model with controlling variables before lockdowns and UFCD Model with controlling variables during lockdowns. The quantification of NO_2_ variation, urban functional fragmentation and the controlling variables for association analysis is shown in Table S1, S2 and S3, respectively. The results of model evaluation are displayed in Table 1, indicating that involving controlling variables, including population, Air Quality Index (AQI), fine particulate matter with a diameter less than 2.5 μm (PM_2.5_), particulate matter with a diameter of less than 10 μm (PM_10_), ozone (O_3_), sulfur dioxide (SO_2_), carbon monoxide (CO), temperature, humidity and wind speed, is effective to depict NO_2_ variation influenced by urban functional fragmentation with higher accuracies. Moreover, accuracies of UFCD Model during lockdowns are higher than UFCB Model before lockdowns, with 0.565 and 0.72 respectively, indicating that more significant impacts of urban functional fragmentation are shown on NO_2_ variations with restricted anthropogenic emissions compared with the impacts with no emission restrictions. In addition, due to the lower accuracies of UFB Model and UFD model, with 0.0807 and 0.107, the influence of lockdown based on models without controlling variables is insignificant.

**Table 1:** Model evaluation.

	$R^{2}$	Deviance explained
UFB Model	0.0807	11.9%
UFD Model	0.107	14.4%
UFCB Model	0.565	64.3%
UFCD Model	0.72	77.4%

$R^{2}$ values of 0.0807, 0.107, 0.565 and 0.72 and deviance explained percentages of 11.9%, 14.4%, 64.3% and 77.4% of UFB, UFD, UFCB and UFCD Models are revealed. Values of $R^{2}$ and deviance explained percentages of UFCB and UFCD Models are much higher than those of UFB and UFD Models.

**Figure 6:**
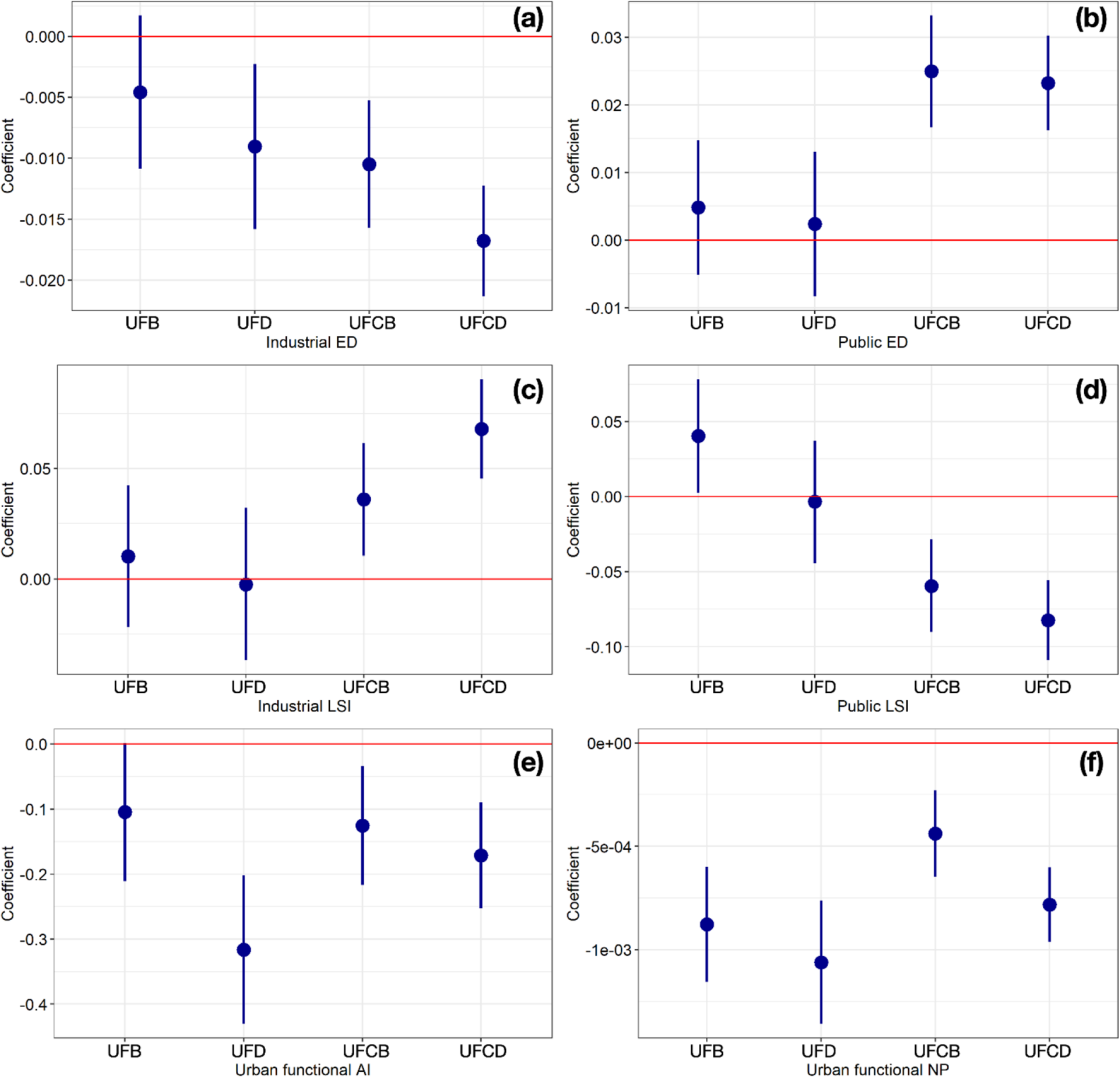
Coefficient changes and 95% CI of urban functional fragmentation characteristics on NO_2_ variations based on four models. (**a**) Industrial ED; (**b**) Public ED; (**c**) Industrial LSI; (**d**) Public LSI; (**e**) Urban functional AI; (**f**) Urban functional NP. One unit of industrial ED is associated with − 0.0046 (95% CI − 0.0109 to 0.0017), − 0.0090 (95% CI − 0.0158 to − 0.0023), − 0.0105 (95% CI − 0.0157 to − 0.0053) and − 0.0168 (95% CI − 0.0213 to − 0.0122) NO_2_ variations of UFB, UFD, UFCB and UFCD models, respectively. Per unit of public ED is linked with 0.0048 (95% CI − 0.0052 to 0.0148), 0.0024 (95% CI − 0.0083 to 0.0131), 0.0249 (95% CI 0.0166 to 0.0332) and 0.0232 (95% CI 0.0162 to 0.0303) NO_2_ variations for four models. NO_2_ variations of 0.0103 (95% CI − 0.0218 to 0.0424), − 0.0024 (95% CI − 0.0370 to 0.0321), 0.0360 (95% CI 0.0105 to 0.0616) and 0.0680 (95% CI 0.0454 to 0.0907) of four models are influenced by per unit of industrial LSI. For the public LSI characteristic, one unit of this is associated with 0.0405 (95% CI 0.0026 to 0.0784), − 0.0035 (95% CI − 0.0443 to 0.0373), − 0.0594 (95% CI − 0.0902 to − 0.0287) and − 0.0825 (95% CI − 0.1092 to − 0.0558) NO_2_ variations of four models. Per unit of urban functional AI is associated with − 0.1047 (95% CI − 0.2110 to 0.0016), − 0.3165 (95% CI − 0.4308 to − 0.2022), − 0.1253 (95% CI − 0.2167 to − 0.0338) and − 0.1712 (95% CI − 0.2524 to − 0.0899) NO_2_ variations of four models. One unit of urban functional NP is associated with − 0.0009 (95% CI − 0.0012 to − 0.0006), − 0.0011 (95% CI − 0.0014 to − 0.0008), − 0.0004 (95% CI − 0.0006 to − 0.0002) and − 0.0008 (95% CI − 0.0010 to − 0.0006) NO_2_ variation of four models.

The impact of urban functional fragmentation was quantified by the 95%-Confidence-Interval (CI) coefficient changes of the UFB, UFD, UFCB and UFCD models, as shown Fig. 6. Due to the higher accuracy of UFCD, the association between urban functional fragmentation and NO_2_ variations was analyzed with controlling variables and lockdown restrictions. Specifically, the decreasing NO_2_ variations, representing the lower-level NO_2_ differential between COVID-19 lockdowns and normal days, are associated with the increasing industrial ED, public LSI, urban functional AI and NP. The lower-level NO_2_ differential indicates the anthropogenic emissions in fragmented industrial and public lands after lockdown. The synthetical urban functional fragmentations also contribute to the NO_2_ emissions despite of the lockdown and social-distancing restriction. The NO_2_ emission during the lockdown can be explained by the energy uses of the factories in industrial land and essential human activities in public land high-mixing urban functions. On the other hand, increasing NO_2_ variations, representing higher-level NO_2_ differential between COVID-19 lockdowns and normal days, are associated with higher values of public ED and industrial LSI. Based on the declining trends of NO_2_ concentrations in COVID-19 lockdowns which are both found in the previous research^16^ and represented in Fig. 3, the NO_2_ differential indicates that the NO_2_ concentrations in lockdowns are much larger than that in normal days. The NO_2_ variation-fragmentation association may due to the reason that higher of public ED and industrial LSI are usually related to large occupation of the green land and small manufacturers with lower energy use.

One should be noted that compared with the major influence of air pollutants and meteorological conditions, only partial impacts of the urban functional fragmentation are revealed. It has been proved by the findings that the accuracies of UFCB and UFCD models are 0.484 and 0.613 higher than UFB and UFD models, respectively. In addition, among all urban functional fragmentation metrics, the highest absolute values of coefficients are attributed by the urban functional AI metric, approximately from 0 to 0.4, while the lowest absolute values of coefficients are revealed in the urban functional NP, approximately from 5e-04 to 1e-03. The discrepancy of the coefficient ranges may be influenced by the different scale among urban functional fragmentation metrics, in which urban functional AI and NP range from 95.35 to 99.99 and from 1–1067, respectively. Despite of the overall lower coefficients and the diverse coefficients among fragmentation metrics, this study retrieved the partial effect of urban functional fragmentation characteristics by controlling the dominant impact of air pollutants and meteorological factors. Findings could provide suggestions for the government to assess the current urban function planning in NO_2_ controlling.

## Discussion

Based on the coefficient comparison between UFCB Model and UFCD Model (Fig. 6), this section discusses the mediated impacts of anthropogenic air pollutant emissions on the urban functional fragmentation-NO_2_ concentration association. As the ${R}^{2}$ values of UFCB and UFCD models with controlling variables are 0.484 and 0.613 higher than UFB and UFD without controlling variables, only UFCB and UFCD models are considered to discuss these mediated impacts. Specifically, the absolute values of coefficients in industrial ED, industrial LSI, public LSI, urban functional AI and urban functional NP are higher than those in the UFCB Model. The higher absolute values of coefficients in the UFCD Model indicate that greater associations are revealed between urban functional fragmentation and NO_2_ variation under the circumstance of anthropogenic-emission restriction. On the other hand, the absolute values of coefficients in public ED in the UFCD Model are lower than those of UFCB Model, which indicates lower association between public ED and NO_2_ variations with the effect of anthropogenic-emission restriction. However, the differences of coefficients between UFCB Model and UFCD Model are all lower than 0.1, which indicates the relative limited impact of anthropogenic-emission restriction. Since the changes of NO_2_ concentration are significantly influenced by the air pollutant and the meteorological factors and solely partially affected by the urban functional fragmentation, the coefficient changes lower than 0.1 are acceptable to evaluate the mediated impact of anthropogenic-emission restriction.

The findings indicate that based on the anthropogenic-emission restriction, greater impacts of industrial, public and synthetical urban functional fragmentation are shown on the NO_2_ variations between COVID-19 and normal days. The greater NO_2_ variations during the anthropogenic-emission restrictions can be explained that emissions from the transportation and commercial functions have been significantly reduced due to the lockdown and social-distancing restriction. In addition, the directions (namely positive or negative trends) of urban functional fragmentation-NO_2_ variation association are not changed by the mediated impact of the anthropogenic-emission restriction.

This study provides an insight into evaluating urban functional fragmentation patterns that are associated with NO_2_ emissions under the circumstance of COVID-19 lockdowns, which could support managers and policymakers to allocate urban resources and public facilities. Specifically, assessing the spatial distribution of industrial and public functions in terms of the degree of fragmentation can be useful for implementing a sustainable environment development strategy. While the influence of transportation on air quality has been restricted during COVID-19 lockdowns, energy uses associated with the heterogenous expanding of industrial and public lands should be concerned. This study has also provided suggestions for the governments which specific fragmentation characteristics can be concerned in a higher priority. For instance, we have found that fragmentation patterns of public LSI show the higher potential to contribute to NO_2_ emissions compared with the public ED.

The government should also be sensitive to the synthetic influence of urban functions and the corresponding fragmentation patterns. The increasing number and aggregation degree of urban function patches may contribute to the demands of accessibility to different public facilities, rendering the potential factors to increase anthropogenic air pollution emissions. Despite that the complexity urban structures and functions can improve urban vitality and socioeconomic development, the negative effect on air quality should be concerned to mitigate public health risk. Accordingly, the governments should keep the balance between urban vitality and air quality caused by urban functional fragmentations to develop sustainable societies.

## Conclusion

This study explored the impact of urban functional fragmentation on NO_2_ variations with anthropogenic-emission restriction in China. Counterfactual NO_2_ concentrations during COVID-19 were predicted based on historical NO_2_ patterns using SARIMAX and were further utilized to quantify the variations comparing with the NO_2_ in normal days using DTW. Then, characteristics including industrial/public ED and LSI and urban functional AI and NP were utilized to depict urban functional fragmentation. Four models, including UFB, UFD, UFCB and UFCD Models based on GAMs are further proposed to investigate the impact of urban functional fragmentations on NO_2_ variations before and during COVID-19 lockdowns.

The results reveal that under the circumstance of anthropogenic-emission restriction, industrial ED, public LSI, urban functional AI and NP are negatively associated with NO_2_ variations, while public ED and industrial LSI are positively related to NO_2_ variations. Compared with the fragmentation-NO_2_ variation association before lockdown, the mediated impact of anthropogenic-emission restriction partially increases the effect of industrial ED, industrial LSI, public LSI, urban functional AI and urban functional NP while decreases the effect of public ED on NO_2_ variation, with the absolute values of coefficients ranging within 0.1. However, the impact of restricted anthropogenic emissions does not change the positive or negative directions of fragmentation-NO_2_ variation association.

Although the proposed research has explored the urban functional fragmentation patterns associated with NO_2_ variation, limitations still exist and are required to be solved in future studies. In quantifying NO_2_ variation, the variations between observed and predicted NO_2_ concentrations are measured. Although the performance of SARIXAM has been evaluated by comparing forecasting results in different time periods, the prediction errors during COVID-19 lockdown cannot be eliminated. As a result, the coefficients of NO_2_ variations could be influenced by these errors. To avoid this issue, regression model will be modified by concerning forecasting biases as controlling variables in further research. Moreover, as only fragmentation characteristics are involved in depicting urban functional morphologies, the spatial heterogeneity and also its dynamics of different functions should also be involved in future studies. In addition, only the potential impact of urban functional fragmentation on NO_2_ variation has been investigated. How to integrate the fragmentation factors with human activities based on the restricted anthropogenic emissions to predict the on-going NO_2_ variation remain an issue to be solved.

## Methods

### Data sources

Daily surface NO_2_ observations from the China National Environmental Monitoring Center (CNEMC) (available at http://106.37.208.233:20035/) were adopted. Considering of the temporal changes of long-term NO_2_, daily surface NO_2_ from Jan. 1st, 2015 to May 1st, 2020 were collected^51^. Specifically, a total number of 145 sites were involved, occupying 27 provinces throughout mainland China. In addition, controlling variables, including population, AQI, PM_2.5_, PM_10_, O_3_, SO_2_, CO, temperature, humidity and wind speed, are also collected. In particular, AQI, PM_2.5_, PM_10_, O_3_, SO_2_, and CO are collected from CNEMC, while temperature, humidity and wind speed are collected in the weather stations from the China National Meteorological Science Data Center (CMA) (available at https://data.cma.cn). It should be noted that as NO_2_ sites and weather stations usually are not located in the same location, weather stations which are nearest to the NO_2_ sites are chosen to quantify the corresponding meteorological data.

For the data source utilized to depict urban functional fragmentation, urban function classification is adopted from urban land use category mapping proposed by Gong, et al.^52^. In particular, level-1 land use classification scheme was utilized, including residential, commercial, industrial, transportation and public management and service, to represent corresponding urban functions.

### Estimation of NO_2_ variation

NO_2_ varies drastically during the lockdowns in COVID-19 because of the restricted urban mobility. In particular, the variation of NO_2_ concentrations can be quantified based on the differences of NO_2_ in lockdowns and NO_2_ in normal days. While NO_2_ concentrations in lockdowns are observed through air stations, NO_2_ concentrations in normal days can be predicted according to the periodical patterns of historical NO_2_ concentrations. A SARIMAX-DTW-based model is proposed to quantify NO_2_ variations in this study. Specifically, counterfactual NO_2_ concentrations during COVID-19 (from Jan. 1st, 2020 to May 1st, 2020) are predicted based on historical NO_2_ using SARIMAX model, with exogenous variables including air temperature, relative humidity and wind speed. The basic SARIMAX model can be presented as follows:

1 $$SARIMAX\left( {p,d,q} \right)\left( {P,D,Q} \right)_{t}$$

in which $p,d,q$ demote the autoregressive order, difference order and moving average order, while $P,D,Q$ indicates the seasonal autoregressive order, difference order and moving average order, respectively. To determine those parameters, the Augmented Dickey-Fuller test and Osborn, Chui, Smith, and Birchenhall test and Canova-Hansen test. In addition, the Ljung–Box test and the Jarque–Bera test are applied to examine the randomness and normality of the time series. The performances of SARIMAX with different parameters are evaluated by Akaike Information Criterion (AIC).

As this study aims on predicting the temporal patterns of NO_2_ during COVID-19 using SARIMAX, under the assumption that no lockdown policies are proposed, historical NO_2_ concentrations are required for model training. In particular, NO_2_ from three different time periods before COVID-19 epidemic are utilized as training data, including NO_2_ (1) from Jan. 1st, 2015 to Nov. 1st, 2019, (2) from Jan. 1st, 2015 to Dec. 1st, 2019 and (3) from Jan. 1st, 2015 to Jan. 1st, 2020. And the corresponding data for NO_2_ prediction in normal days includes NO_2_ (1) from Nov. 1st, 2019 to May 1st, 2020, (2) from Dec. 1st, 2019 to May 1st, 2020, and from (3) Jan. 1st, 2020 to May 1st, 2020. Because of the time inconsistence of COVID-19 outbreaks in different regions in China, the model accuracies are evaluated based on different time period division. Furthermore, to evaluate the proposed SARIMAX models with different time periods, mean observed-predicted NO_2_ errors for each air station are calculated:

2 $$MOPE_{i} = \mathop \sum \limits_{k = 1}^{n} \frac{{\left| {NO_{2obs,k} - NO_{2pre,k} } \right|}}{{NO_{2obs,k} }}/n \cdot 100$$

where $MOPE_{i}$ refers to the mean daily observed-predicted NO_2_ error of the *i*th air station. $NO_{2obs,k}$ and $NO_{2pre,k}$ represent the observed NO_2_ concentrations and the predicted NO_2_ concentrations of the *k*th day, respectively. $n$ is the total number of days during time periods. Specifically, time period with lower values of ${MOPE}_{i}$ is selected for SARIMAX modelling.

To investigate the effects of restricted urban mobility during COVID-19, variations between the actual and counterfactual NO_2_ concentrations are required to be quantified. Moreover, NO_2_ variation should be matched with optional alignment instead of day-to-day matching to reduce prediction errors of SARIMAX model. Thus, the DTW model is utilized to fit the non-linear patterns of NO_2_ concentrations. NO_2_ variation between the actual and counterfactual time periods are calculated as follows:

3 $$DTW\left( {T_{act} ,T_{cou} } \right) = \mathop {\arg \min }\limits_{{W = w_{1} \ldots w_{k} \ldots w_{K} }} \sqrt {\mathop \sum \limits_{{k = 1,w_{k} = \left( {i,j} \right)}}^{K} \left( {t_{act,i} - t_{con,j} } \right)^{2} }$$

where $T_{act}$ and $T_{con}$ refer to the actual and counterfactual temporal NO_2_ concentrations during COVID-19 epidemic. *W* represents the warping path aligned by $T_{act}$ and $T_{con}$. Higher $DTW\left( {T_{act} ,T_{cou} } \right)$ values denote higher degree of NO_2_ variation.

### Urban functional fragmentation metrics

This study measures urban functional fragmentation from two perspectives, including single-urban functional fragmentation and overall urban functional fragmentation, as shown in Table 2. For the single-urban functional fragmentation, industrial and public functions are considered as they are highly associated with anthropogenic air pollutant emissions^33,34^. In particular, as the urbanization process may change the monotonous patterns to heterogenous distribution such as the emerging of small areas of parks and hospitals, the complexities of industrial and public function patches can effectively exhibit varying trends of anthropogenic energy uses (such as the increasing transportation). Research has revealed the potential relationship between impervious LSI and the edge characteristics of public spaces and NO_2_ distribution^22,53^. This study adopts metrics including industrial/public ED and LSI to depict the fragmentation characteristics in terms of patch complexity.

**Table 2:** Indicators for depicting urban functional fragmentation.

	Indicator	Equation	Definition	Fragmentation application
Single-urban functional fragmentation depiction	Industrial/Public Edge Density (ED)	$ED = \frac{{\mathop \sum \nolimits_{k = 1}^{m} e_{ik} }}{A}$	$e_{ik}$ indicates the total length of edge of a certain patch *k* belonging to the *i*th class. *m* indicates the total number of patches of the *i*th class. *A* refers to the total areas belonging to the *i*th urban functional class	As most of the anthropogenic emissions are generated from industrial and public lands, the shape complexities depicted by ED and LSI of industrial and public urban functional patches are considered. Higher values of *ED* and *LSI* indicate higher degree of edge density and complexity, respectively
	Industrial/Public Landscape Shape Index (LSI)	$LSI = \frac{{0.25\mathop \sum \nolimits_{k = 1}^{m} e_{ik} }}{\sqrt A }$		
Synthetical urban functional fragmentation depiction	Urban functional Aggregation Index (AI)	$AI = \left[ {\mathop \sum \limits_{i = 1}^{m} \left( {\frac{{g_{ii} }}{{\max \left( {g_{ii} } \right)}}} \right)P_{i} } \right] \cdot \left( {100} \right)$	$g_{ii}$ represent the number of like adjacencies between pixels of the patches of the *i*th class. $\max \left( {g_{ii} } \right)$ refers to the maximum number of $g_{ii}$. $P_{i}$ denotes the percentage of the *i*th urban functional class within each 3 km-radium area. *N* refers to the total number of urban functional patches within individual buffers	For all-type urban functions, higher values of NP represent large total number of urban functional patches and higher fragmentation degree within individual buffers. The AI values increase as the urban functional patches within buffers are increasingly aggregated
	Urban functional Number of Patches (NP)	$NP = N$		

For the overall urban functional fragmentation, the changes of the regular patterns of human activities (such as the synthetic influence of manufacturing, residential and transportation) that lead to the anthropogenic-emission variation are considered. To depict these specific fragmentation characteristics, the possible increased number of urban function patches and their connections are utilized. Liang, et al.^54^ have discussed the effect of AI landscape characteristics of urban forms on NO_2_ concentrations, while characteristics of patch number and density in metropolitan regions have been evaluated with the influence on air quality^55^. Thus, this study adopts AI and NP metrics for quantifying synthetic urban functional patterns.

**Figure 7:**
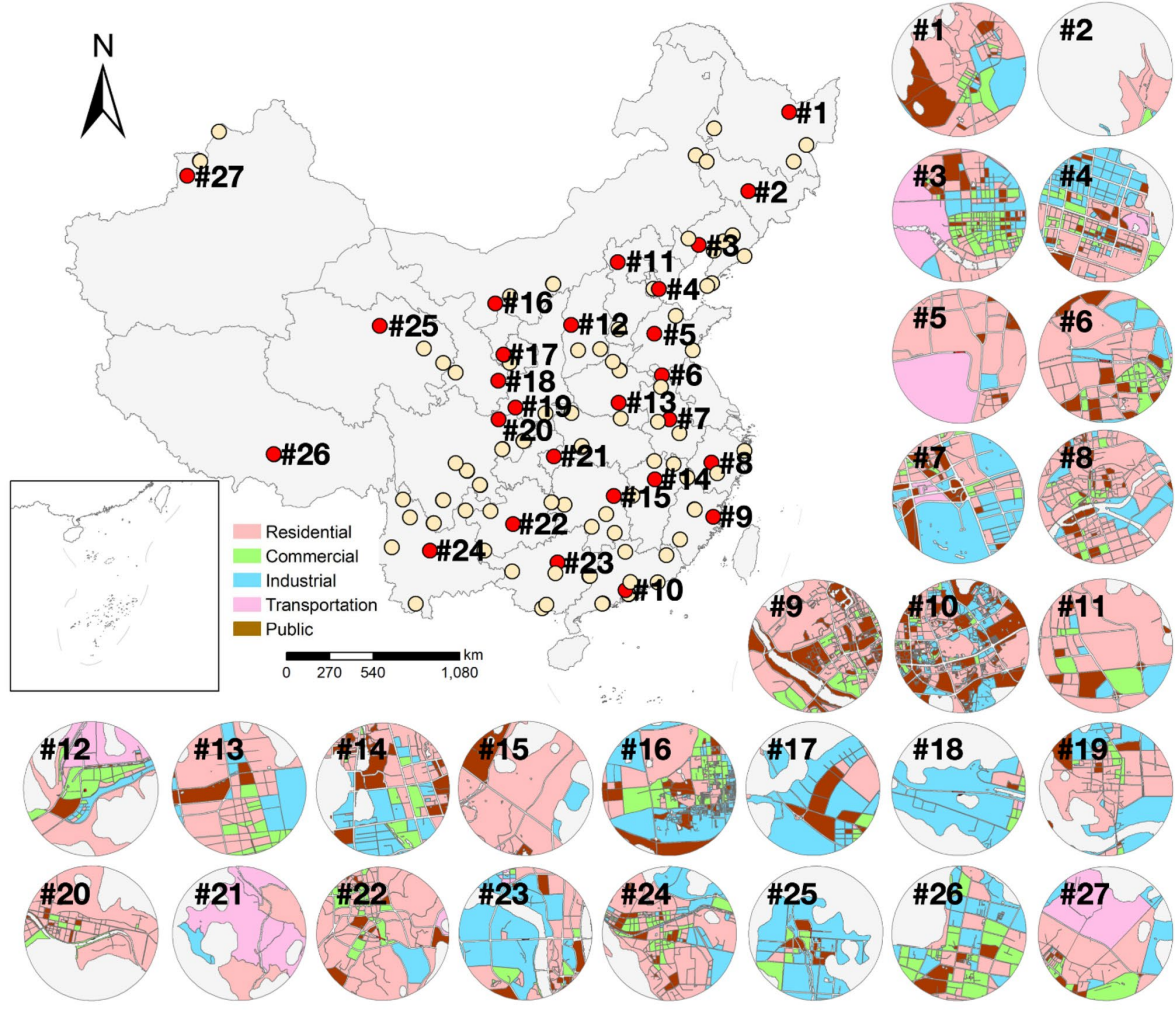
Distribution of urban functions within buffers of selected air stations. 27 sites of 145 air stations with 3 km buffers were displayed. Urban functions including residential, commercial, industrial, transportation and public functions are involved to depict fragmentation characteristics. The maps are performed using ArcGIS Pro software (version 2.7, https://www.esri.com/en-us/arcgis/products/arcgis-pro/overview).

To depict urban functional fragmentation characteristics based on the proposed metrics, continuous areas around air station are extracted as station-sensed regions. In particular, the buffer of each air station within 3 km is chosen, as previous studies have revealed that radius of air stations within approximately 2.5 km are highly correlated with NO_2_ concentrations^56,57^. On this basis, 3 km-radium areas of 145 air stations were extracted. Among the 145 air stations, 27 air stations were randomly chosen for displaying the urban function distributions. As shown in Fig. 7, the proportions of urban functional occupation vary among different cities.

### Statistical analysis

The effects of urban functional fragmentation on NO_2_ variation before and during lockdowns (lockdown date is selected as Jan. 24, 2020) during COVID-19 are analyzed using GAMs. Specifically, the proposed GAMs are implemented based on Gaussian distribution. NO_2_ variation are utilized as dependent variable while six urban functional fragmentation characteristics, including industrial ED, public ED, industrial LSI, public LSI, urban functional AI and urban functional NP, are considered as independent variables. In addition, possible confounding effects, including population, AQI, PM_2.5_, PM_10_, O_3_, SO_2_, CO, temperature, humidity and wind speed, are involved as controlling variables. The average daily values of AQI, PM_2.5_, PM_10_, O_3_, SO_2_, CO, temperature, humidity and wind speed are adopted while the total population within the 3-km buffers around the air stations is utilized. Then, four comparative models are designed, concerning additional environmental factor for model controlling and different scenarios, namely before and during COVID-19 lockdown^16,58^ (Table 3): (1) UFB Model, including urban functional fragmentation characteristics before lockdowns; (2) UFD Model, including urban functional fragmentation characteristics during lockdowns; (3) UFCB Model, including both urban functional fragmentation characteristics and controlling variables before lockdowns; and (4) UFCD Model, including urban functional fragmentation characteristics and controlling variables during lockdowns. The proposal GAMs in UFB Model and UFD Model are defined as Eq. (4) and GAMs in UFCB Model and UFCD Model are defined as Eq. (5), respectively:

4 $$\log E\left( {Y_{i} } \right) = \alpha + \beta_{1} Ind_{EDi} + \beta_{2} Pub_{EDi} + \beta_{3} Ind_{LSIi} + \beta_{4} Pub_{LSIi} + \beta_{5} UF_{AIi} + \beta_{6} UF_{NPi}$$

5 $$\begin{aligned} \log E\left( {Y_{i} } \right) & = \alpha + \beta _{1} Ind_{{ED_{i} }} + \beta _{2} Pub_{{ED_{i} }} + \beta _{3} Ind_{{LSI_{i} }} + \beta _{4} Pub_{{LSI_{i} }} \\ & \quad + \beta _{5} UF_{{AI_{i} }} + \beta _{6} UF_{{NP_{i} }} + s\left( {Pop_{i} } \right) + s\left( {AQI_{i} } \right) + s\left( {PM_{{25_{i} }} } \right) \\ & \quad + s\left( {PM_{{10_{i} }} } \right) + s\left( {O_{{3i}} } \right) + s\left( {CO_{i} } \right) + s\left( {SO_{{2i}} } \right) \\ & \quad + s\left( {Tem_{i} } \right) + s\left( {Hum_{i} } \right) + s\left( {Wind_{i} } \right) \\ \end{aligned}$$

**Table 3:** Independent and dependent variables for GAMs.

Variables		UFB Model	UFD Model	UFCB Model	UFCD Model
		Before lockdown	During lockdown	Before lockdown	During lockdown
Independent variables	Industrial ED	✓	✓	✓	✓
	Public ED	✓	✓	✓	✓
	Industrial LSI	✓	✓	✓	✓
	Public LSI	✓	✓	✓	✓
	Urban functional AI	✓	✓	✓	✓
	Urban functional NP	✓	✓	✓	✓
Controlling variables	Population			✓	✓
	AQI			✓	✓
	PM_2.5_ (${\upmu }$ g/m^3^)			✓	✓
	PM_10_ (${\upmu }$ g/m^3^)			✓	✓
	O_3_ (${\upmu }$ g/m^3^)			✓	✓
	CO (mg/m^3^)			✓	✓
	SO_2_ (${\upmu }$ g/m^3^)			✓	✓
	Temperature (°C)			✓	✓
	Humidity (%)			✓	✓
	Wind speed (m/s)			✓	✓
Dependent variables	NO_2_ variation	✓	✓	✓	✓

where $E\left( {Y_{i} } \right)$ is the expected NO_2_ differences of the *i*th air station during COVID-19. $\alpha$ and $\beta$ are the intercept and regression coefficient, respectively. $Ind_{EDi}$, $Pub_{EDi}$, $Ind_{LSIi}$, $Pub_{LSIi}$, $UF_{AIi}$ and $UF_{NPi}$ are the fragmentation characteristics depicting urban functions including industrial ED, public ED, industrial LSI, public LSI, urban functional AI and urban functional NP of the *i*th air station. $Pop_{i}$, $AQI_{i}$, $PM_{25i}$, $PM_{10i}$, $O_{3i}$, $SO_{2i}$, $CO_{i}$, $Tem_{i}$, $Hum_{i}$ and $Wind_{i}$ represent the controlling variables including population, AQI, PM_2.5_, PM_10_, O_3_, SO_2_, CO, temperature, humidity and wind speed of the *i*th air station, respectively. $s(variable)$ refers to the smoother function of a specific variable based on the penalized smoothing spline, with the degree of freedom evaluated by Akaike Information Criterion (AIC).

## Supplementary Information

Supplementary Information.

## Abbreviations

NO_2_: Nitrogen Dioxide
AQI: Air Quality Index
PM_2.5_: Fine particulate matter with a diameter less than 2.5 μm
PM_10_: Particulate matter with a diameter of less than 10 μm (PM_10_),
O_3_: Ozone
SO_2_: Sulfur Dioxide
CO: Carbon Monoxide
COVID-19: Novel coronavirus disease 2019
SARIMAX-DTW: Autoregressive Integrated Moving Average with external variables-Dynamic Time Warping
ED: Edge Density
LSI: Landscape Shape Index
AI: Aggregation Index
NP: Number of Patches
PLADJ: Percentage of Like Adjacencies
NP: Number of Patches
COHESION: Patch Cohesion Index
UFB: Urban functional Fragmentation characteristics Before lockdowns
UFD: Urban functional Fragmentation characteristics During lockdowns
UFCB: Urban functional Fragmentation characteristics and Controlling variables Before lockdowns
UFCD: Urban functional Fragmentation characteristics and Controlling variables During lockdowns
GAMs: Generalized Additive Models
VIF: Variance Inflation Factor
CI: Confidence-Interval
CNEMC: China National Environmental Monitoring Center
CMA: China National Meteorological Science Data Center
AIC: Akaike Information Criterion
