# Supplementary material for: Effects of urban functional fragmentation on nitrogen dioxide (NO2) variation with anthropogenic-emission restriction in China
Source: Sci Rep. 2021 Jun 7;11:11908. doi: 10.1038/s41598-021-91236-w (PMC8184851; doi:10.1038/s41598-021-91236-w)
Supplement: Supplementary file 1 — Supplementary Information. [file 41598_2021_91236_MOESM1_ESM.pdf]

Supplementary Information (SI) for

Effects of Urban Functional Fragmentation on Nitrogen Dioxide (NO_2) Variation with Anthropogenic-emission Restriction in China

Yuan Meng [a], Man Sing Wong [a,b,*], Hanfa Xing [c,d], Rui Zhu [a] , Kai Qin [e], Mei-Po Kwan [f,g], Kwon Ho Lee [h], Yin Tung Kwok [a] and Hon Li [a]

[a] *Department of Land Surveying and Geo-Informatics, The Hong Kong Polytechnic University, Hong Kong; myuan.meng@connect.polyu.hk; rui.zhu@smart.mit.edu; yt-coco.kwok@connect.polyu.hk; honli.li@polyu.edu.hk*
[b] *Research Institute for Sustainable Urban Development, The Hong Kong Polytechnic University, Hong Kong*
[c] *School of Geography, South China Normal University, Guangzhou, Guangdong, China; xinghanfa@sdnu.edu.cn*
[d] *College of Geography and Environment, Shandong Normal University, Jinan, Shandong, China*
[e] *School of Environment and Spatial Informatics, China University of Mining and Technology, Xuzhou, China; qinkai@cumt.edu.cn*
[f] *Department of Geography and Resource Management, and Institute of Space and Earth Information Science The Chinese University of Hong Kong, Hong Kong; mpk654@gmail.com*
[g] *Department of Human Geography and Spatial Planning, Utrecht University, 3584 CB Utrecht, The Netherlands*
[h] *Department of Atmospheric & Environmental Sciences, Gangneung-Wonju National University, Gangneung 25457, South Korea; khlee@gwnu.ac.kr*

*Correspondence: ls.charles@polyu.edu.hk; Tel.: +852-3400-8959

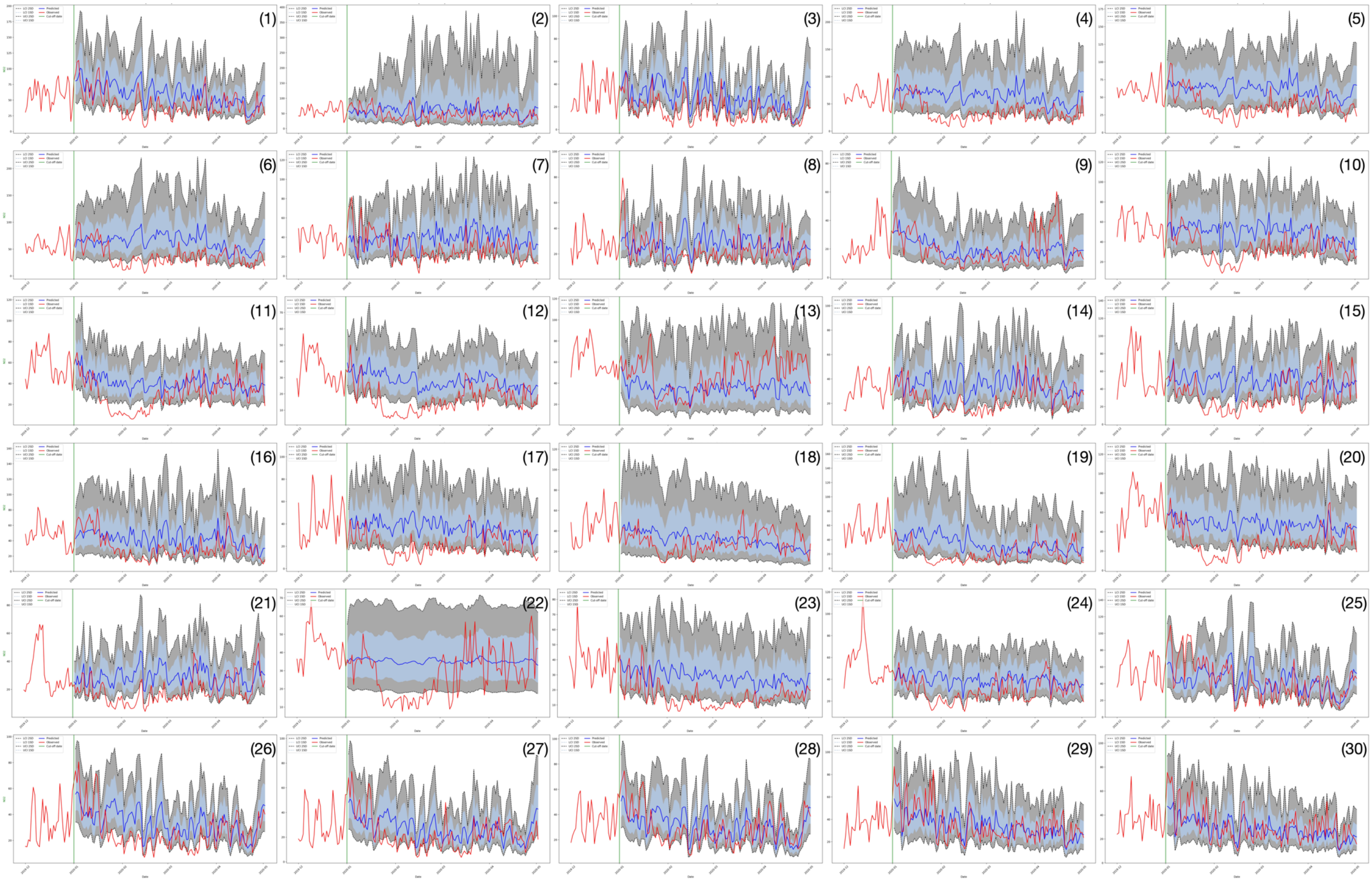

Fig. S1. Predicted NO2 concentrations of 145 air stations using SARIMAX during Jan. 1st, 2020 to May 1st, 2020.

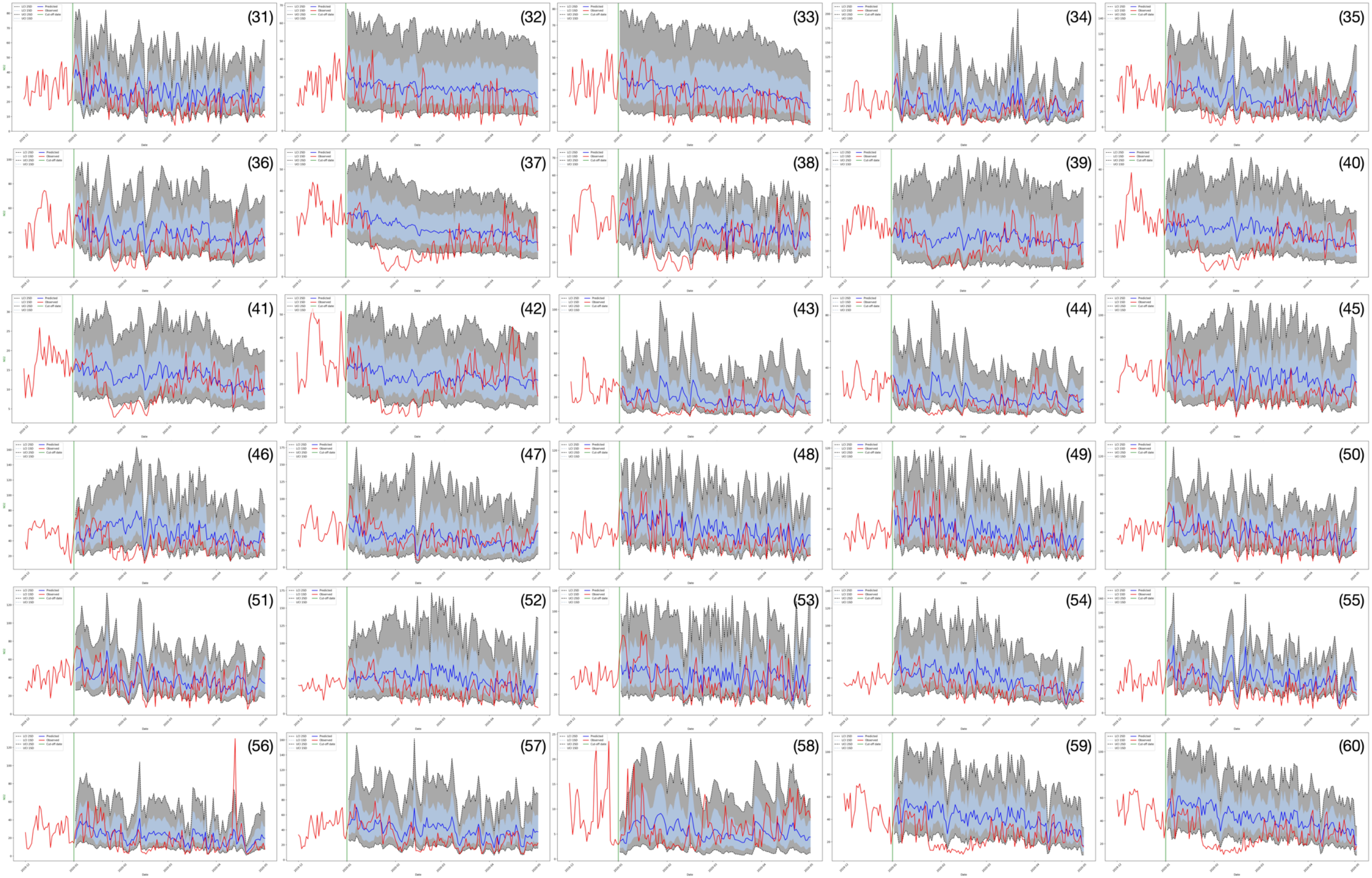

Figure S1. Continued.

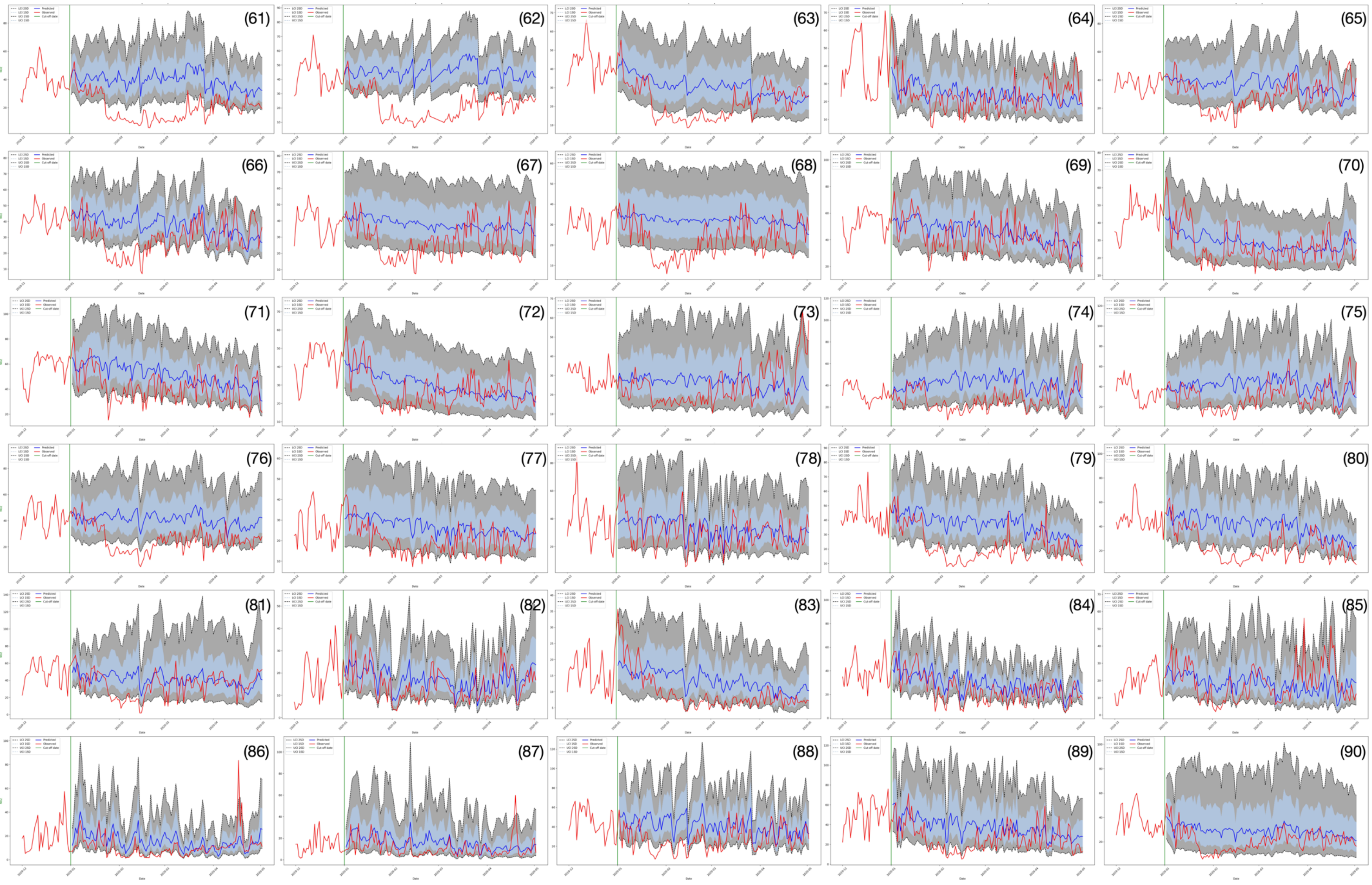

Figure S1. Continued.

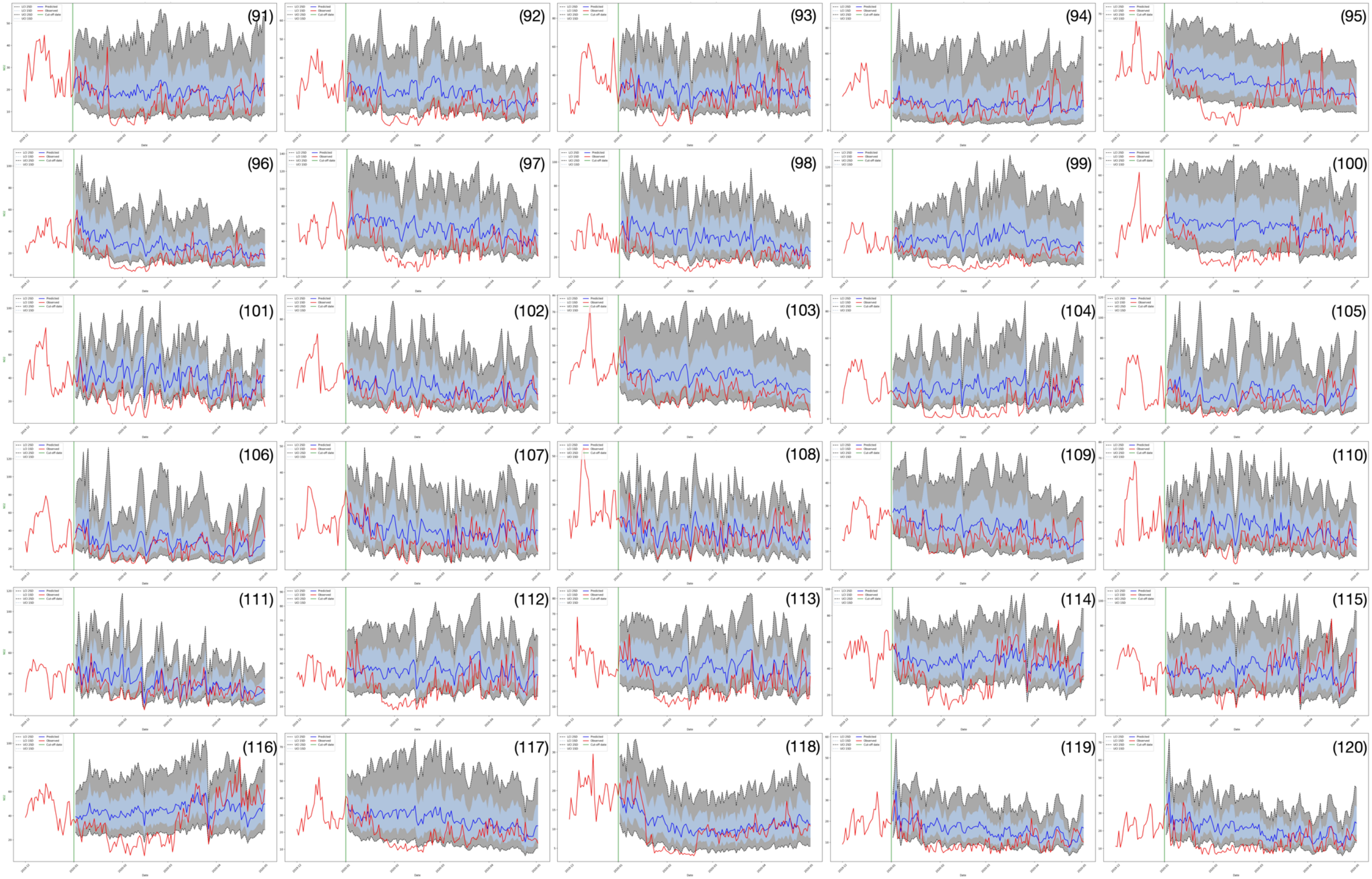

Figure S1. Continued.

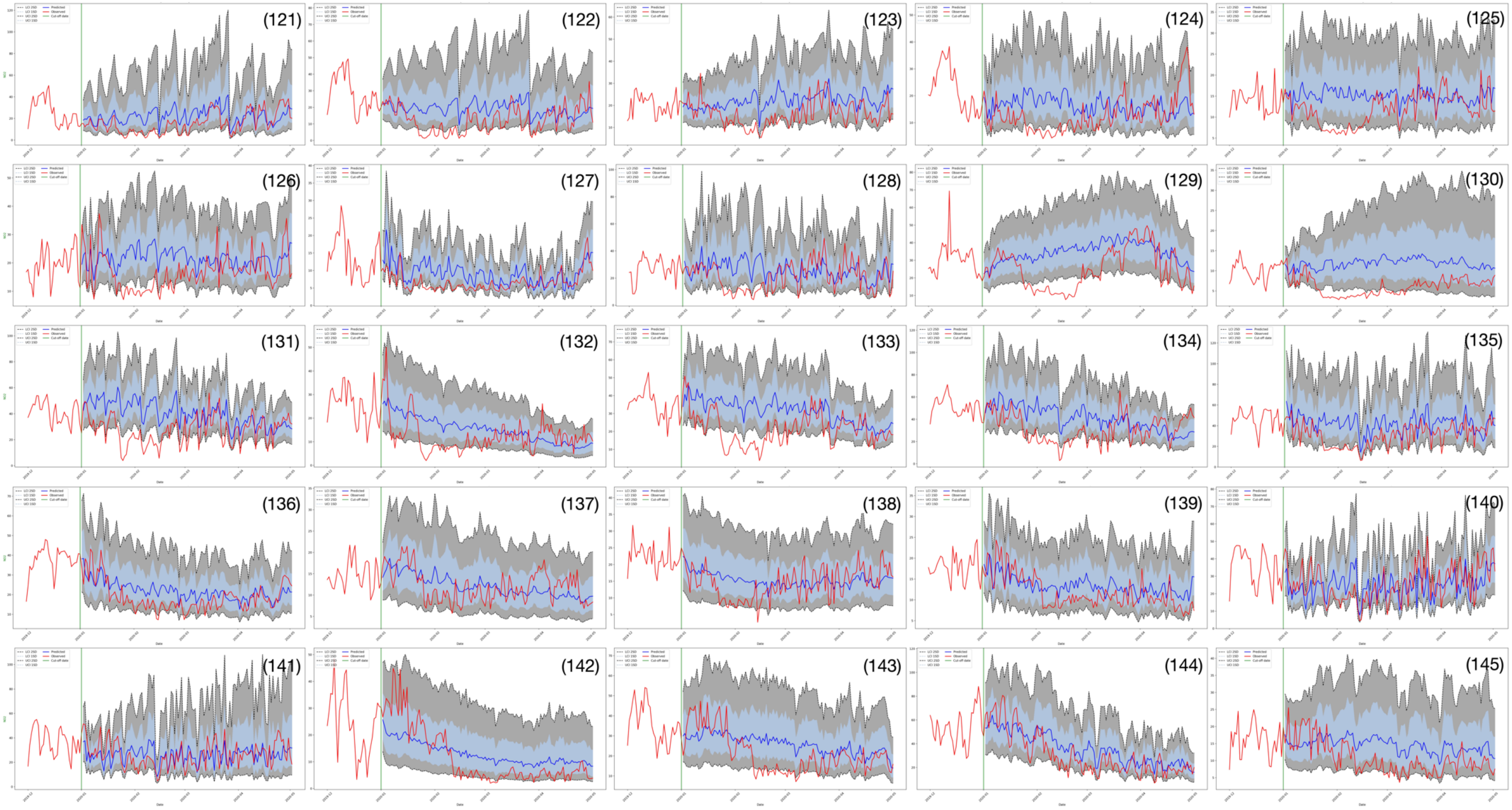

Figure S1. Continued.

Table S1. Time-series NO_2 variations between observed and predicted NO_2 of 145 air stations before and during lockdown.

Site	NO_2 variation before lockdown	NO_2 variation during lockdown
1	1696.586402	367.4569134
2	1864.785396	395.9169916
3	1081.521625	214.993166
4	3228.431139	394.680297
5	2515.367473	289.5494277
6	3014.249032	324.8251128
7	1093.569658	363.7177852
8	704.1517231	293.1399453
9	640.8594809	129.4358985
10	1280.971583	229.6175323
11	1325.241843	207.5733083
12	1109.690243	246.094284
13	1204.152355	349.8409151
14	765.4764539	228.3871429
15	1597.125713	285.0843033
16	1301.348985	280.738594
17	1499.680645	280.2316986
18	858.0407145	198.3514793
19	1751.838271	356.7428042
20	1766.898169	344.8646357
21	1036.998894	214.8575166
22	1353.952492	215.0855623
23	784.0679123	304.7416264
24	1019.727763	157.6680746
25	1032.083736	586.972472
26	985.5578209	353.8073724
27	788.8670266	285.5568269
28	741.4337374	326.375914
29	791.4481187	305.2332074
30	668.7688532	367.6179064
31	621.9867593	184.2199512
32	707.0924064	181.8244939
33	734.2355088	180.6579677
34	1290.653864	242.3261874
35	1489.437838	361.2257716
36	904.0182705	267.6123943
37	1089.004762	164.5224768
38	888.803687	224.255508
39	324.0769466	46.14505944

40	632.7126887	110.6349847
41	360.1508737	73.51315405
42	724.5985332	187.5828794
43	863.465255	119.0222335
44	733.5035047	129.4093797
45	939.5131988	310.9002992
46	1535.60033	364.293399
47	862.1380963	336.599213
48	1074.696646	279.9401007
49	1036.699992	300.8647618
50	1093.329633	314.0003857
51	1086.644298	337.8560649
52	1322.096743	306.6883687
53	1096.78027	365.4733055
54	900.0654757	279.9817935
55	1386.44451	319.7015094
56	787.0811023	255.0377302
57	866.1888813	195.3673314
58	265.1893806	75.01266016
59	1474.451248	291.1532146
60	1470.299729	282.1711869
61	2277.064855	196.6096677
62	3168.862776	251.6307147
63	1168.954141	164.2383891
64	589.3923643	151.9447899
65	1116.54335	121.9685815
66	1200.412196	172.4446904
67	1509.193615	176.3685725
68	1156.650239	181.5972018
69	773.4385405	182.0987817
70	702.0685521	223.4739129
71	1151.973422	238.3056461
72	736.7440499	184.4335802
73	911.2557015	109.2825867
74	1334.091991	143.7783492
75	1182.841171	132.5934827
76	1597.364049	184.508793
77	712.1097011	173.8909314
78	786.0242693	312.8571899
79	1571.517287	164.3132586
80	1657.944605	218.7443518
81	1126.424741	207.4139096
82	378.527273	143.9453145

83	277.141	120.8847766
84	694.5557523	191.2279677
85	658.7583845	165.7782434
86	577.9332718	155.0124119
87	509.499948	188.5728895
88	1274.359209	334.4978185
89	1305.167521	254.6950697
90	949.7143524	179.2535261
91	575.1392801	116.9729452
92	866.3581041	127.0507711
93	906.9376484	202.619467
94	770.0248188	84.59693152
95	1200.652409	228.1423477
96	1073.222234	272.6961662
97	2024.121566	316.7079625
98	1715.290075	322.2684585
99	2096.130664	308.8390787
100	1324.594364	158.631453
101	1242.081429	244.5731812
102	728.4727453	131.0801574
103	745.2473522	115.4597181
104	997.0104899	166.561556
105	826.5610317	230.2151628
106	931.6684875	249.2316721
107	391.4086008	63.89401428
108	326.7939511	84.21101856
109	423.8615516	141.3899285
110	633.7661296	169.6096118
111	795.996902	209.0722495
112	1264.923922	198.0442921
113	1230.579888	125.9307618
114	1258.457192	178.3182071
115	971.7316901	117.8310703
116	1411.097462	274.6933412
117	1072.73893	150.3299295
118	293.8083123	88.61716547
119	627.8142777	148.4086581
120	846.0260031	185.0212904
121	779.8379774	124.0485649
122	826.460038	127.6450371
123	521.8185991	66.59501057
124	551.2606941	90.65847065
125	469.2517757	83.61693586

126	611.9566565	157.9073029
127	205.4923886	81.1199835
128	1010.366414	147.154345
129	1021.03913	70.5002583
130	748.4915646	73.39016298
131	1325.16163	280.9450005
132	640.5266143	219.3188155
133	1064.987717	229.8175497
134	1192.233705	225.290185
135	1184.620238	274.7968364
136	325.776672	127.6984796
137	258.7186631	58.5917336
138	398.4107504	80.43200248
139	209.935757	66.04186361
140	706.4282529	185.145278
141	667.9226404	215.7355907
142	343.8854038	200.8075057
143	450.562523	187.1781626
144	557.5902372	244.7121729
145	303.5853835	87.50818756

Table S2. Urban functional fragmentation characteristics of 145 air stations.

Site	Industrial ED	Industrial LSI	Public ED	Public LSI	Urban functional NP	Urban functional AI
1	15.7757	4.6832	6.9542	3.3315	92	98.871
2	39.59	10.9004	14.5384	7.5944	268	97.2995
3	3.7958	2.4077	6.2218	3.3916	84	98.9354
4	25.3892	5.4197	6.8701	3.0676	41	99.0332
5	25.3892	5.4197	6.8701	3.0676	41	99.0332
6	16.8143	4.295	10.6144	3.1973	41	99.0023
7	11.8966	7.6549	37.1529	12.8273	578	95.8264
8	18.5999	5.6905	8.3016	5.9302	78	98.1473
9	1.377	3.3462	0.1274	1.0909	17	99.7132
10	9.6514	4.0341	10.0797	4.867	174	98.205
11	17.3796	6.9109	15.0326	8.6433	349	97.0064
12	7.904	5.3348	20.2644	6.4968	167	98.0697
13	27.5587	6.2116	15.1004	6.7523	139	98.1866
14	-	-	43.9467	13.5546	311	96.9693
15	24.356	6.9865	16.304	6.4625	183	98.061
16	1.108	1.1219	3.7734	3.9167	37	99.1851
17	16.9332	7.1523	25.2574	7.8628	157	97.5168
18	9.5791	5.9266	30.0679	9.5333	181	97.7652

19	61.7474	19.5962	51.1385	13.978	804	95.3513
20	11.6559	4.6792	22.6428	7.4243	106	98.6331
21	2.5523	1.3578	6.3259	4.3355	118	98.0894
22	29.0179	8.4815	15.6266	6.0164	195	97.9114
23	24.3814	7.259	6.4175	3.545	123	98.2933
24	3.0197	1.9837	5.3278	3.061	53	99.0988
25	23.3148	6.2699	4.7684	2.8672	83	98.7157
26	18.7912	5.7746	4.0091	2.3028	66	98.9604
27	34.1536	8.3314	6.709	3.9748	85	98.6705
28	23.3148	6.2699	4.7684	2.8672	83	98.7157
29	51.9119	10.7306	12.5983	6.6163	120	98.2129
30	51.9119	10.7306	12.5983	6.6163	120	98.2129
31	20.7916	4.5251	2.1559	2.2402	31	98.7947
32	53.3035	13.2374	24.4	9.6649	258	96.9293
33	53.3035	13.2374	24.4	9.6649	258	96.9293
34	0.7452	1.711	13.6052	5.4331	116	98.1458
35	11.2526	6.0494	6.9661	3.6357	191	97.6097
36	21.9145	5.3148	1.4245	2.133	99	98.8866
37	9.384	3.6835	12.5563	3.7627	246	98.2968
38	13.1339	4.1048	11.1765	3.4728	152	98.4543
39	20.9143	4.2525	-	-	36	99.0738
40	15.5571	3.9202	-	-	35	99.1439
41	15.5571	3.9202	-	-	35	99.1439
42	59.5466	13.9054	24.0197	6.9592	333	97.2653
43	51.5303	12.4725	25.5351	7.3986	418	96.9265
44	58.3603	12.7018	23.3091	8.7574	351	96.9053
45	3.1411	2.2278	5.7053	3.6306	19	99.447
46	3.1411	2.2278	5.7053	3.6306	19	99.447
47	10.4317	4.4712	17.73	5.0662	64	98.6575
48	3.2334	5.6608	12.6222	6.1494	147	98.2082
49	3.0837	4.4286	10.6026	5.8501	95	98.6158
50	6.2657	5.0716	4.3382	4.2328	84	98.6333
51	6.9059	5.6838	2.8947	4.829	66	99.1766
52	6.9059	5.6838	2.8947	4.829	66	99.1766
53	6.9059	5.6838	2.8947	4.829	66	99.1766
54	6.9059	5.6838	2.8947	4.829	66	99.1766
55	17.0576	6.223	10.8148	4.9837	246	97.6965
56	7.1025	3.9331	10.1591	4.5836	106	98.8441
57	38.8035	8.075	5.3482	4.2563	93	97.9902
58	-	-	-	-	1	99.9879
59	19.5277	5.3573	6.9211	2.3498	89	98.6974
60	22.1505	5.043	5.573	2.6257	85	98.8073
61	15.1906	5.6667	12.5565	4.0869	131	98.0485

62	6.3561	3.0103	14.9506	5.7177	88	98.3737
63	20.3279	5.5552	4.9855	4.4551	98	98.9972
64	29.6157	6.0715	7.1738	4.9524	165	98.4117
65	-	-	-	-	1	99.9879
66	-	-	-	-	1	99.9879
67	29.7301	9.7746	6.0851	5.4608	168	97.804
68	29.7301	9.7746	6.0851	5.4608	168	97.804
69	29.7301	9.7746	6.0851	5.4608	168	97.804
70	29.7301	9.7746	6.0851	5.4608	168	97.804
71	28.7436	11.3424	6.0124	5.3856	166	97.7567
72	29.7301	9.7746	6.0851	5.4608	168	97.804
73	18.4113	4.8507	4.0694	4.1458	122	98.5672
74	14.1565	4.3627	7.3234	5.9798	123	98.5614
75	18.4113	4.8507	4.0694	4.1458	122	98.5672
76	10.5309	3.8749	13.9115	5.0341	66	98.6341
77	3.0286	2.7873	10.7721	4.7394	63	98.7874
78	3.4879	3.1711	9.7788	4.8427	66	98.8529
79	17.5046	4.8324	10.6931	5.895	226	97.9685
80	16.9916	4.5863	15.9757	6.6025	213	98.0749
81	8.5122	5.2293	6.7178	4.9171	125	98.5423
82	36.6353	9.4469	27.0238	7.4969	150	97.576
83	53.4334	13.168	20.3441	8.0605	498	96.9796
84	9.7331	4.0603	4.3895	3.0422	48	98.9239
85	15.6079	5.3164	11.4413	3.651	75	98.4471
86	12.5298	3.7754	13.006	4.0958	45	99.0255
87	8.2553	3.4956	11.8908	4.0717	96	98.3512
88	-	-	-	-	22	99.6054
89	22.3056	5.845	9.7684	7.302	145	98.0145
90	26.8531	7.1168	26.5645	9.6288	226	97.3693
91	21.3735	9.7109	3.1201	4.0642	106	98.8843
92	16.8474	8.1285	4.1096	5.114	101	98.9651
93	15.608	4.5827	6.4202	3.4618	106	98.4474
94	8.1036	5.2808	2.4977	3.3224	129	97.8977
95	0.9823	2.0859	5.237	2.3454	104	98.4972
96	9.1794	8.526	17.8985	8.6375	290	97.7232
97	13.6005	4.4985	6.9976	3.6014	120	98.1301
98	9.1569	3.4032	6.5181	3.2773	88	98.5486
99	1.2285	1.6909	0.4815	2.4659	56	98.6895
100	3.2709	2.4444	0.0389	1.4545	19	99.2505
101	11.6427	8.8295	6.6596	5.4195	284	97.4097
102	16.3327	5.9384	3.7876	2.7436	108	98.6367
103	8.1416	5.358	10.0173	5.9031	180	98.3632
104	3.6904	2.4085	4.0604	2.4713	46	98.9293

105	8.1812	4.9102	1.2173	2.0117	63	98.9662
106	10.4607	4.7793	0.4423	2.1552	56	99.2245
107	11.2774	4.2371	0.2053	2	24	99.4341
108	6.902	4.367	6.8047	4.995	168	97.8475
109	17.2951	6.0229	5.6494	4.0611	956	98.9585
110	19.6631	4.4235	7.0281	3.5024	71	99.0783
111	0.1558	1.6296	5.0145	5.9259	101	98.5629
112	4.3027	5.6511	3.8533	2.9945	52	99.437
113	7.4683	5.7025	0.9254	2.0072	69	98.9997
114	7.2112	3.3544	2.3743	2.684	31	99.4824
115	7.8324	3.2698	0.8899	2.3197	81	99.2479
116	8.9754	2.8496	1.0991	2.7584	70	99.4434
117	6.8187	5.5665	-	-	39	99.4057
118	-	-	-	-	1	99.988
119	1.8399	2.3188	1.2773	1.7783	84	98.5172
120	3.5779	2.8162	1.8403	2.4645	78	98.3626
121	5.0317	4.6292	2.7423	3.5227	33	99.1491
122	3.0395	2.9873	0.4175	2.6522	29	99.2482
123	-	-	0.7133	1.2857	9	99.8081
124	5.0677	2.2897	1.4408	1.4908	20	99.4162
125	32.9612	8.5974	17.7915	5.013	141	98.1738
126	19.4611	6.4175	9.8376	5.2612	93	98.2728
127	24.8126	10.0178	18.7803	10.3544	1607	97.3055
128	19.1581	6.9789	12.1727	7.5361	283	97.5188
129	5.6443	4.5063	5.4567	7.798	221	98.1375
130	23.7549	7.6814	4.4576	3.9366	144	98.7621
131	16.4616	5.2869	4.2486	2.8429	98	98.4601
132	3.9087	4.0719	5.8993	5.319	84	98.716
133	1.9873	3.4057	6.5157	4.7744	104	98.2902
134	28.0813	5.7729	0.7998	1.7769	36	99.207
135	21.6206	5.8958	8.8712	5.2946	54	98.8058
136	12.3228	4.6235	3.6528	4.3279	24	99.4064
137	7.1413	5.0576	7.8278	4.0439	29	99.4513
138	14.634	4.4762	0.1558	1.76	25	99.384
139	31.6654	7.2982	5.6772	4.1554	64	98.9773
140	35.2914	7.0077	4.6289	3.1604	65	98.7276
141	27.1367	6.0347	9.5848	3.7222	50	98.9928
142	18.6554	7.2336	7.7485	4.3186	74	98.8598
143	30.9552	7.5654	9.1169	4.8935	93	98.4449
144	8.1543	4.1654	3.9621	4.875	59	98.6505
145	23.8242	7.5246	11.7201	4.431	509	98.5361

Table S3. Controlling variables of 145 air stations.

Site	AQI	$PM_{2.5}$ (μg/m³)	O_3 (μg/m³)	PM_{10} (μg/m³)	SO_2 (μg/m³)	CO (mg/m³)	Temperature (°C)	Humidity (%)	Wind speed (m/s)	Population
1	100.81	67.09	55.61	111.70	23.19	1.38	14.12	52.51	2.19	67192
2	93.19	63.54	53.52	100.83	20.14	1.24	14.18	56.09	2.49	20171
3	68.05	33.73	70.48	87.14	19.94	0.77	9.39	47.26	2.80	23062
4	128.36	90.89	57.54	155.45	37.21	1.58	15.17	56.41	2.96	168270
5	131.71	90.22	54.10	164.81	45.57	1.49	15.06	56.31	2.96	168270
6	126.25	86.41	58.31	156.86	45.94	1.60	15.12	56.37	2.96	328777
7	65.48	39.41	67.95	70.27	21.48	0.97	12.03	60.75	2.96	588269
8	62.55	38.93	79.95	60.04	17.16	0.76	11.75	64.93	3.14	127123
9	72.09	46.92	68.58	68.42	10.53	0.87	6.18	64.83	2.87	10173
10	99.41	64.21	65.93	120.24	34.10	1.11	15.87	65.83	1.71	589165
11	66.55	44.89	59.99	66.66	14.19	0.80	19.24	71.66	1.73	248806
12	51.49	33.14	55.78	50.00	10.52	0.69	19.44	72.26	1.08	146794
13	90.68	62.33	50.41	93.48	15.03	0.90	16.86	75.31	2.05	6241
14	48.22	26.71	57.34	52.00	6.43	0.72	20.76	73.52	2.24	204197
15	71.02	40.93	56.83	85.86	18.79	1.13	19.04	73.50	1.72	10408
16	105.73	68.31	77.62	132.72	28.94	3.45	15.37	55.21	2.39	14469
17	46.01	27.56	66.47	45.25	7.82	0.68	23.48	78.86	2.58	91632
18	47.23	29.23	58.78	46.47	7.66	0.68	23.47	78.90	2.59	167256
19	49.40	31.10	56.94	48.12	12.25	0.80	23.32	77.74	1.84	34966
20	56.04	36.68	61.90	53.07	14.95	0.81	23.23	76.34	2.31	1903
21	59.28	38.35	54.90	63.06	15.43	0.74	15.11	80.39	2.44	256201
22	54.76	31.26	48.60	63.00	19.01	0.95	9.81	36.45	1.92	141701
23	51.30	22.48	69.01	55.47	9.15	0.86	10.00	36.99	1.94	116199
24	76.10	51.18	52.05	84.65	17.95	0.80	18.82	77.08	1.70	146666
25	86.49	48.75	62.13	105.91	28.09	1.21	7.75	46.03	2.35	69984
26	66.69	27.02	78.84	83.11	24.20	0.81	7.79	46.14	2.35	67412
27	60.93	28.24	84.83	71.78	18.73	0.79	7.78	46.08	2.35	36225

28	62.67	26.36	75.97	75.14	15.50	0.74	7.75	46.08	2.35	69984
29	66.51	39.14	79.00	69.10	18.95	0.91	10.14	63.20	3.51	93940
30	68.42	43.68	75.95	68.02	16.19	0.80	10.13	63.23	3.52	93940
31	61.98	38.40	58.82	67.47	28.09	1.19	9.34	67.39	2.66	61222
32	58.03	37.55	58.59	60.22	24.33	1.08	17.67	76.49	2.12	74318
33	64.70	40.83	55.44	69.97	28.10	1.22	17.62	76.44	2.12	74318
34	79.43	50.28	69.51	91.38	18.73	0.81	13.88	67.05	2.89	71658
35	91.11	57.50	76.59	108.98	42.69	1.08	14.25	57.59	2.01	91774
36	57.80	37.45	49.69	59.51	20.77	1.13	20.58	79.05	2.34	44653
37	43.55	27.46	49.59	42.30	7.92	0.90	21.98	76.15	1.57	74575
38	47.67	30.25	49.33	48.17	8.00	0.93	21.99	76.21	1.57	68953
39	41.37	24.61	74.55	40.13	8.59	0.78	23.32	77.60	2.36	82860
40	42.62	24.77	72.19	42.70	9.64	0.76	23.31	77.60	2.36	135386
41	43.02	25.72	72.60	42.53	9.15	0.73	23.29	77.55	2.36	135386
42	48.40	30.56	55.92	48.08	9.79	0.86	23.05	80.75	3.43	16131
43	48.49	31.21	67.14	44.96	7.61	0.77	23.20	81.46	3.40	202579
44	48.55	31.87	59.96	46.51	7.44	0.92	23.23	81.50	3.40	262360
45	93.67	62.50	66.60	109.50	39.67	1.83	10.83	57.20	3.17	63173
46	91.71	60.57	71.07	103.60	34.85	1.68	10.79	57.13	3.17	63173
47	97.37	64.45	57.25	113.81	49.53	1.89	14.53	51.99	1.51	258539
48	76.72	49.36	67.20	85.28	34.46	1.35	11.22	49.32	2.46	176666
49	72.65	47.40	67.59	78.65	29.71	1.20	11.21	49.26	2.46	94166
50	76.19	47.87	52.94	90.85	34.94	1.50	8.96	59.39	2.16	432068
51	78.74	51.62	46.04	90.77	33.86	1.78	9.00	59.45	2.16	292126
52	70.58	44.82	49.22	72.63	40.97	1.56	8.99	59.46	2.16	292126
53	61.92	37.55	59.48	63.12	24.39	1.23	8.94	59.42	2.16	292126
54	68.97	40.70	50.78	77.23	13.37	1.13	9.06	59.54	2.15	292126
55	80.13	51.26	68.34	81.72	52.13	1.04	10.90	51.82	2.51	547425
56	56.97	34.03	55.10	56.48	15.06	0.67	4.78	58.62	2.42	255012
57	61.84	36.53	44.45	67.51	17.42	0.70	4.96	63.33	3.16	509260

58	46.75	23.80	103.30	39.53	14.26	0.55	12.46	80.37	3.50	32941
59	104.93	69.21	65.33	117.47	22.27	1.26	15.96	60.38	2.21	40537
60	102.00	68.25	65.45	111.17	21.43	1.14	15.88	60.34	2.21	17854
61	86.01	59.23	48.95	93.61	15.96	0.93	16.66	75.88	1.85	428841
62	87.77	61.26	45.43	92.93	13.55	1.06	16.74	75.85	1.85	324121
63	84.97	58.29	47.10	89.00	14.04	0.95	17.41	72.31	1.46	81813
64	69.09	49.23	59.93	69.15	26.90	1.17	20.92	74.23	2.27	353682
65	73.00	51.22	43.94	68.76	19.62	0.96	18.49	79.01	1.62	266116
66	80.42	57.42	45.57	81.42	16.30	0.88	18.42	79.04	1.61	209389
67	74.09	50.95	53.08	76.81	16.57	0.95	21.36	55.61	1.62	127443
68	79.32	55.15	51.83	79.59	17.57	0.94	21.36	55.65	1.62	127443
69	60.19	34.60	49.64	69.44	40.17	1.77	21.34	55.49	1.62	127443
70	57.47	34.13	53.47	63.46	40.78	1.51	21.36	55.47	1.62	127443
71	58.51	33.15	44.04	67.40	34.65	1.47	21.39	55.53	1.62	144162
72	49.90	28.82	50.95	53.83	20.97	1.32	21.36	55.57	1.62	127443
73	72.89	51.08	49.22	77.39	9.86	0.74	17.82	77.72	1.81	143337
74	77.29	53.06	46.69	85.98	13.29	0.78	17.81	77.70	1.81	128438
75	73.07	51.63	52.71	75.75	12.42	0.89	17.83	77.69	1.81	143337
76	89.69	55.94	49.94	105.69	11.03	1.05	14.48	52.18	1.50	280326
77	71.51	29.56	81.68	94.09	14.46	0.63	10.79	43.99	2.20	123179
78	84.83	38.47	71.22	113.76	55.79	0.87	10.77	44.00	2.20	124856
79	84.42	56.93	69.67	92.99	12.95	0.83	16.28	71.12	2.06	236214
80	88.26	58.84	65.26	101.97	11.71	0.79	16.24	71.17	2.06	220346
81	85.09	53.69	43.74	104.16	51.03	1.62	10.74	53.31	1.90	71010
82	51.71	27.31	62.64	55.65	9.13	0.62	6.19	45.72	2.39	196491
83	64.99	32.67	95.61	79.46	13.71	0.65	9.16	34.64	2.07	40082
84	75.32	43.20	64.43	84.59	29.53	1.47	9.77	48.33	2.74	185419
85	61.22	37.26	64.91	64.32	12.68	0.56	5.72	53.47	2.68	226629
86	43.24	22.80	62.17	45.19	12.23	0.66	4.49	61.30	3.93	150533
87	50.78	31.76	59.86	52.32	9.81	0.76	2.00	61.89	2.45	134543

88	75.92	52.45	57.24	77.98	24.13	1.00	16.96	79.91	2.59	45178
89	91.58	63.67	65.99	92.90	20.76	0.84	15.29	72.53	2.17	215878
90	73.56	47.77	66.32	82.66	10.61	0.70	16.25	73.83	1.85	190712
91	38.93	24.86	48.63	35.14	9.23	0.84	20.41	75.59	1.50	193980
92	39.82	23.50	48.07	38.27	9.26	0.81	20.34	75.65	1.49	150938
93	45.00	24.30	45.78	47.70	9.93	0.74	20.65	74.83	1.78	49783
94	57.11	37.21	46.55	61.80	9.13	0.78	18.45	76.22	1.02	278960
95	62.97	42.95	51.04	65.05	21.93	1.00	17.97	77.95	1.78	221456
96	58.42	38.41	58.51	62.67	28.84	0.70	18.20	77.03	1.65	147110
97	107.60	72.96	59.88	128.60	31.49	1.40	15.60	58.86	2.11	509666
98	92.94	62.50	74.26	104.54	16.12	0.81	15.23	70.14	2.12	83182
99	73.19	49.09	46.90	76.76	15.99	1.14	16.05	69.34	1.69	362017
100	63.36	42.43	32.66	64.36	9.32	0.90	16.96	78.61	0.88	107119
101	70.40	48.53	51.54	73.27	16.73	1.11	18.80	75.04	1.62	568427
102	56.35	32.37	52.45	63.66	17.47	1.16	17.68	81.04	3.38	231069
103	63.84	42.92	47.20	66.52	18.29	0.76	18.35	80.64	2.26	109062
104	70.24	40.05	50.66	84.10	16.14	1.14	17.17	80.38	1.20	88364
105	54.71	36.66	50.70	55.30	13.80	1.20	21.53	79.27	1.99	201246
106	50.37	31.83	45.61	51.66	13.29	0.85	21.56	79.33	2.00	157376
107	48.38	30.50	51.61	49.59	8.46	0.87	22.54	82.17	2.46	75517
108	52.90	33.64	57.90	55.19	17.53	1.10	22.55	80.94	2.84	145968
109	56.51	36.83	46.42	58.01	14.80	0.87	22.47	77.05	1.53	125379
110	61.62	41.41	53.00	63.08	17.08	0.92	21.13	78.39	1.81	73384
111	53.27	27.23	51.97	58.45	16.72	0.81	16.04	68.64	1.74	36513
112	71.73	49.69	54.47	71.27	12.51	0.85	18.18	73.88	1.22	144284
113	73.02	50.90	49.16	75.17	12.88	0.93	18.22	73.83	1.22	235078
114	69.79	47.51	49.05	75.79	8.59	0.87	17.91	76.72	1.21	206430
115	73.42	51.65	42.01	74.18	11.50	1.02	17.90	76.75	1.21	300590
116	76.43	51.82	48.59	85.00	10.72	0.97	17.89	76.72	1.21	80193
117	57.04	36.19	58.27	59.52	12.28	0.76	16.73	78.21	0.92	125853

118	30.11	13.06	52.41	26.28	7.11	0.42	9.23	61.23	1.23	707
119	39.29	22.48	66.83	37.99	15.70	0.82	17.93	55.31	1.43	271967
120	40.54	23.46	69.13	38.43	19.09	0.84	17.96	55.45	1.42	261994
121	51.81	26.14	44.24	57.21	8.98	0.90	17.03	77.23	1.38	86308
122	54.60	27.55	47.59	62.35	12.02	0.81	17.01	77.20	1.38	87649
123	45.76	29.17	49.87	44.79	13.36	0.93	13.59	79.74	1.09	59752
124	40.33	19.82	47.44	41.33	13.00	0.83	16.95	79.48	1.87	166546
125	43.20	26.93	63.75	39.90	7.02	0.72	17.46	64.54	1.58	169382
126	49.70	24.86	76.26	50.82	24.83	0.87	12.59	73.07	1.63	187794
127	36.54	15.74	71.74	33.81	10.15	0.78	13.84	58.12	2.64	52003
128	38.20	21.67	53.72	36.82	18.19	0.72	17.15	63.30	2.17	192490
129	55.43	32.46	35.24	61.66	6.24	0.91	23.50	73.40	0.85	67255
130	29.03	13.50	54.88	25.21	9.10	0.73	6.51	64.54	2.67	7891
131	78.06	51.71	50.18	85.12	14.55	1.30	15.60	73.80	1.09	257759
132	57.02	36.19	63.30	58.03	9.36	0.67	16.25	70.53	1.18	193547
133	70.91	47.11	48.49	75.49	20.27	1.09	16.28	70.49	1.19	349971
134	71.27	41.87	60.40	84.52	16.39	0.84	12.25	63.32	1.95	108505
135	65.39	33.05	57.20	77.15	13.95	0.74	9.67	59.68	2.21	258671
136	58.90	32.41	70.30	67.74	15.93	0.93	3.35	63.93	1.76	28088
137	62.92	35.74	74.30	73.22	14.99	0.82	7.13	50.74	1.99	7586
138	55.72	28.45	81.15	61.93	12.49	0.64	5.29	48.45	1.60	33021
139	55.89	24.83	88.38	66.03	16.88	0.73	4.85	38.04	1.72	37165
140	75.78	35.05	64.39	95.93	12.63	0.88	7.87	54.03	2.32	58194
141	65.77	31.18	65.79	79.29	8.65	0.78	7.88	54.03	2.32	15713
142	52.20	23.73	61.05	59.22	9.79	0.69	7.08	62.41	1.67	21283
143	60.50	31.83	51.91	71.10	16.89	0.96	7.01	62.51	1.67	75459
144	78.44	48.10	56.54	84.76	16.63	1.53	10.57	60.97	1.43	157737
145	40.40	15.61	69.61	42.27	6.37	1.08	8.11	58.05	1.77	69228
